# Assessing Genotypic and Environmental Effects on Endophyte Communities of *Fraxinus* (Ash) Using Culture Dependent and Independent DNA Sequencing

**DOI:** 10.3390/jof7070565

**Published:** 2021-07-15

**Authors:** Anindita Lahiri, Brian R. Murphy, Trevor R. Hodkinson

**Affiliations:** Botany Department, School of Natural Sciences, Trinity College Dublin, The University of Dublin, Dublin D2, Ireland; alahiri@tcd.ie (A.L.); murphb16@tcd.ie (B.R.M.)

**Keywords:** ash, barcoding, communities, core microbiome, dieback, DNA, endophyte, *Fraxinus excelsior*, genotype, next generation sequencing

## Abstract

*Fraxinus excelsior* populations are in decline due to the ash dieback disease *Hymenoscyphus fraxineus*. It is important to understand genotypic and environmental effects on its fungal microbiome to develop disease management strategies. To do this, we used culture dependent and culture independent approaches to characterize endophyte material from contrasting ash provenances, environments, and tissues (leaves, roots, seeds). Endophytes were isolated and identified using nrITS, LSU, or *tef* DNA loci in the culture dependent assessments, which were mostly Ascomycota and assigned to 37 families. Few taxa were shared between roots and leaves. The culture independent approach used high throughput sequencing (HTS) of nrITS amplicons directly from plant DNA and detected 35 families. Large differences were found in OTU diversity and community composition estimated by the contrasting approaches and these data need to be combined for estimations of the core endophyte communities. Species richness and Shannon index values were highest for the leaf material and the French population. Few species were shared between seed and leaf tissue. PCoA and NMDS of the HTS data showed that seed and leaf microbiome communities were highly distinct and that there was a strong influence of *Fraxinus* species identity on their fungal community composition. The results will facilitate a better understanding of ash fungal ecology and are a step toward identifying microbial biocontrol systems to minimize the impact of the disease.

## 1. Introduction

Plants live in association with various microorganisms in the above ground phyllosphere and below ground rhizosphere [1]. These can be endophytic, rhizospheric, and/or epiphytic and can act as beneficial, neutral, or detrimental associates to their host plant [2,3,4,5]. Therefore, it is important to accurately identify the fungal communities found in ash, *Fraxinus excelsior*, if we are to understand how they interact with the dieback pathogen *Hymenoscyphus fraxineus*, which is devastating European populations of this important forest tree [6]. Furthermore, accurate identification of its microbiome organisms is required to investigate the ecological and physiological roles they play in ash trees in general [7,8] and evaluate their potential for the biocontrol of pests and diseases [6,9].

DNA barcoding-based studies are required for community studies of ash fungi because identification using only morphological characters is not adequate for most species [10,11]. Identification is complicated by hybridisation [12], cryptic speciation [13,14], and convergent evolution [15] and is particularly problematic in species rich lineages (5). A further complication is that, in many cases, the anamorph and teleomorph of the same species has been given separate taxonomic names [16]. Standard DNA barcoding involves PCR followed by Sanger DNA sequencing of specific DNA loci [17,18,19]. The Consortium for the Barcode of Life [20] recommends the internal transcribed spacer region (ITS) of nuclear ribosomal DNA (nrDNA) for the identification of fungi and other regions or nuclear ribosomal DNA such as the large and small subunits [21]. Several other loci are also used for fungal identification such as the *RPB1* and *RPB2* subunits of RNA polymerase [22], translation elongation factor 1-alpha (*tef*1) [23,24], the beta-tubulin region (*tub2*/*BenA*) [25], and the mini-chromosome maintenance protein (*MCM7*) [26].

The so called ‘next generation sequencing’ methods were developed in the mid 2000s and marked the beginning of high-throughput sequencing (HTS) of fungal communities [27]. These methods have been improved to offer higher read length and efficient metabarcoding and community analyses [27,28]. Plant associated metagenomic [1,29,30] methods are particularly suitable for unculturable taxa [31] and some studies have investigated the differences of microbiome community composition among host plants, biogeographical pattern, and the temporal succession of the microbiota [32,33,34,35]. Studies of rhizosphere HTS data have also included analyses of mycorrhizal symbionts including ectomycorrhizal and endomycorrhizal fungi [30,36,37,38].

Several studies have been published on the ash microbiome in recent years. Some have studied healthy ash in different environments [39,40] and assessed how the microbiome of ash changes with dieback disease infection [41,42,43,44,45]. Other studies have assessed the fungal microbiome in *F. excelsior* [46] or other *Fraxinus* species in relation to different levels of disease resistance [47,48].

Despite these studies, we need to know more about the ash microbiome in different environments and the influence of genotype on ash endophyte composition. Therefore, we isolated fungal endophytes from a diverse range of *Fraxinus excelsior* provenances and from other related ash species using leaves, roots, and seeds. We aimed to obtain comprehensive taxon lists to study the community level composition in these differing environments and plant tissues. We utilised plants from a European provenance trial of *F. excelsior* growing in a common garden plantation in Ireland (that allowed comparison of contrasting genotypes growing in the same environment), some samples from wild French populations, and different taxa of *Fraxinus* growing at the National Botanic Garden, Glasnevin, Ireland (different *Fraxinus* species in the same environment). This was a novel sampling approach for fungal endophyte community assessments because the ash populations were genotypically diverse and varied at a geographic and environmental level. 

We also directly compared the culturable fungal species associated with ash with those recorded by direct high-throughput (HTS) of nrITS amplicons. The specific objectives were to: (1) isolate and identify a large sample of culturable ash endophytes from *F. excelsior* and related taxa; (2) sequence the nrITS barcoding region and identify them and/or define operational taxonomic units (OTUs); (3) sequence the ash microbiome directly using HTS (Illumina High Seq) of nrITS to assess the unculturable fungal community of the material and how it compares to the culturable component; and (4) compare the endophytes found in a range of different plant tissues including leaves, roots and seeds. 

## 2. Materials and Methods

### 2.1. Sampling

Sampling was undertaken in September 2015. Collection of the leaf and seed samples for endophyte isolation was undertaken from *Fraxinus excelsior* at a provenance trial for forestry research in Roosky, County Roscommon, which included approximately 10-year-old material, collected from 11 countries: Belgium, Czech Republic, Denmark, Germany, France, Ireland, Italy, Lithuania, the Netherlands, the UK, and Poland. All plants had been grown from root-trainer pots in 2005 and 2006 before being planted out in 2007. Trees were then planted on a grey brown podzolic soil with plant spacing at 2 m × 2 m. No fertilizer was used and the site was a moderately open space surrounded by farmer’s fields, hedgerows, and some small areas of forestry. Other species of *Fraxinus* were sampled from mature trees, at least 25 years old, located in the National Botanic Gardens, Glasnevin (*F. americana*, *F. angustifolia*, *F. dipetala*, *F. glabra*, *F. mandshurica*, *F. numidica*, *F. ornus*, *F. pennsylvanica*, *F. potamophila*, *F. pubinervis*, *F. texensis*, *F. xanthoxyloides*). Leaves were also sampled from a wild population of mature *F. excelsior* at Lac d’Annecy, Auvergne-Rhône-Alpes, France and roots sampled from an *F. excelsior* field genebank plantation at Teagasc, Kinsealy, Co., Dublin, Ireland. Fine roots were sampled at approximately two metres from the base of the trunks (1.5 cm in length). A full list of materials is provided in Supplementary Tables S1 and S2.

Endophytes were isolated using fresh plant tissue and the surface sterilisation technique outlined in Murphy et al. [49] and cultured on either malt extract agar with vegitone supplement (MEA; Sigma, Gillingham, Dor., UK, 38954) or MEA media digested with *Fraxinus excelsior* leaves (MEA + F) prior to autoclaving following [6,49]. Different leaf tissue types were compared including lamina, apex, primary vein, and rachis (Supplementary Figure S1). 

The collection of leaf and seed samples for direct HTS of nrITS amplicons was undertaken at the same time as the collections for endophyte isolations (September 2015) from the provenance trial for forestry research in Roosky, County Roscommon; from the National Botanic Gardens, Glasnevin, Dublin, Ireland; and Lac d’Annecy in Auvergne-Rhône-Alpes, France (Supplementary Table S2). After collection, leaf samples were kept inside Ziplock bags filled with silica gel for drying and preservation before DNA extraction (following [50,51]). Seeds were kept in a brown envelope and placed inside the drying cabinet to remove excess moisture. 

### 2.2. Fungal and Plant DNA Extraction and Sequencing

For the culture dependent assessments, a total of 410 isolates were obtained from leaf and root tissues and used for DNA barcoding identification using the nrITS, nrLSU, and *tef* regions. DNA was extracted from the 410 pure cultures of fungal endophytes using a DNeasy Plant Mini Kit from Qiagen (Hilden, NRW, Germany). Approximately 1/8th of the fungal plate hyphal tissue was transferred using sterile forceps and scalpels into a 1.5 mL microcentrifuge tube with a sterile metal bead for homogenisation and tissue disruption using a mixer mill (Retsch MM 300; Haan, Germany) for 1 min at 10 frequency/second (turning the block on the other side and running it for another minute). The rest of the DNA extraction followed the Qiagen manufacturer’s protocol. The final extract of 50 µL was stored at −20 °C until further use. Fifty-five of the isolates were extracted with a hot CTAB extraction (modified from [50,52]). 

For the culture independent assessments, DNA extraction for HTS amplicon sequencing was undertaken from 70 plant samples (Supplementary Table S2). Total DNA from leaflets and seed were extracted by a modified hot CTAB method [50] and purified using the Jetquick DNA Purification Kit (Thermo Fisher Scientific; Loughborough, Leics, UK).

#### 2.2.1. DNA Sequencing and Identification of Pure Culture Fungal DNA

DNA extracts of the isolated fungal DNA were used for amplification in a total volume of 25 µL using ultrapure water, dNTPs (Invitrogen; Thermo Fisher Scientific) 5 × Buffer (Promega; Madison, WI, USA), MgCl_2_ (Promega), *Taq* polymerase (Promega), forward and reverse primers, and 1µL of DNA (approximately 100 ng/µL^−1^). PCR amplification thermal cycling parameters and primers are given in Lahiri [53] and Supplementary Table S3. DNA amplification was confirmed with agarose gel electrophoresis using gel red nucleic acid stain (Biotium 41003; Fremont, CA, USA). Exosap was used to clean-up the PCR reactions prior to cycle sequencing and sequencing following the manufacturer’s protocol. DNA was sequenced with an Applied Biosystems Big Dye Terminator v3.1 Kit, cleaned up with an Applied Biosystems X Terminator Kit (Thermo Fisher Scientific) and run on an Applied Biosystems 3100xl Genetic Analyzer (following manufacturer’s protocols). Approximately half of the samples were sent for commercial sequencing (Source Biosciences; Dublin, Ireland). Sequence trace files were edited in Geneious (v.6; Biomatters) or MEGA 5 [54], and then used in a BLAST search to find the closest matches (percent identity) in GenBank (NCBI). These matches were often 100% and usually above 99.8%. Synonymy and accepted names follow Species Fungorum (http://www.indexfungorum.org/; last accessed on 10 June 2021). 

#### 2.2.2. High-Throughput Sequencing of nrITS-1 Directly from Leaf and Seed

High throughput amplicon sequencing was undertaken on the ITS1 spacer region using the primers: ITS1 5′GGAAGTAAAAGTCGTAACAAGG3′; ITS2-2043R 5′GCTGCGTTCTTCATCGATGC3′. All PCR reactions were carried out with Phusion^®^ High-Fidelity PCR Master Mix (New England Biolabs; Hitchin, Herts., UK). PCR products were mixed in equidensity ratios. Then, mixed PCR products were purified with a Qiagen Gel Extraction Kit (Qiagen, Hilden, Germany). Amplicon sequencing was carried out by Novogene on 70 samples using an Illumina High Seq 250 paired end platform and 50,000 raw tags. The libraries were generated with a NEBNext^®^ UltraTM DNA Library Prep Kit for Illumina and quantified via Qubit before analysis by the Illumina High Seq 250 platform.

Paired-end reads were assigned to samples based on their unique barcode and truncated by cutting off the barcode and primer sequence. Paired-end reads were merged using FLASH (V1.2.7, http://ccb.jhu.edu/software/FLASH/; accessed on 1 September 2019). Quality filtering on the raw tags was performed under specific filtering conditions to obtain the high-quality clean tags according to the Qiime (v.1.7) quality-controlled process. The tags were compared with the Unite reference database (https://unite.ut.ee/; accessed on 1 September 2019) using the UCHIME algorithm to detect chimeric sequences that were subsequently removed to obtain the effective tags.

### 2.3. OTU Cluster, Species Determination, and Diversity Analyses of HTS Data

Sequence analyses were performed using the Uparse software (Uparse v.7.0.1001 http://drive5.com/uparse/; accessed on 1 September 2019) using all the effective tags. Sequences with ≥97% similarity were assigned to the same OTU for the HTS data (following [36]). Representative sequences for each OTU were screened for further annotation. Sequence analyses were performed by BLAST with Qiime (v.1.7) and the UNITE database for species annotation at each taxonomic rank. OTU abundance data were normalised using a standard of sequence number corresponding to the sample with the least sequences. Subsequent analysis of alpha diversity and beta diversity were all performed based on this output normalised data.

Alpha diversity was represented by (1) observed species richness and (2) Shannon index, calculated with Qiime (v.1.7) and displayed with the R software ggplot2 package (v.2.15.3); the significance of pairwise species richness and Shannon index values was assessed with the Tukey and Wilcoxon tests. Beta diversity analysis was also used to evaluate differences between samples in species complexity. Beta diversity on both weighted and unweighted unifrac [55] distances were calculated in QIIME (v.1.7) and significance of all pairwise comparisons assessed with the Tukey and Wilcoxon tests. Principal coordinate analysis (PCoA) was performed with a distance matrix of unweighted unifrac distances among samples. Non-metric multi-dimensional scaling (NMDS) performed with Bray Curtis distances, 100 iterations (stress 0.18). PCoA and NMDS with analyses were performed in QIIME (v.1.7) and displayed with the ggplot2 package in R software. 

## 3. Results

### 3.1. Identification of Cultured Fungal Isolates from Fraxinus Excelsior and Other Fraxinus Species

**Figure 1:**
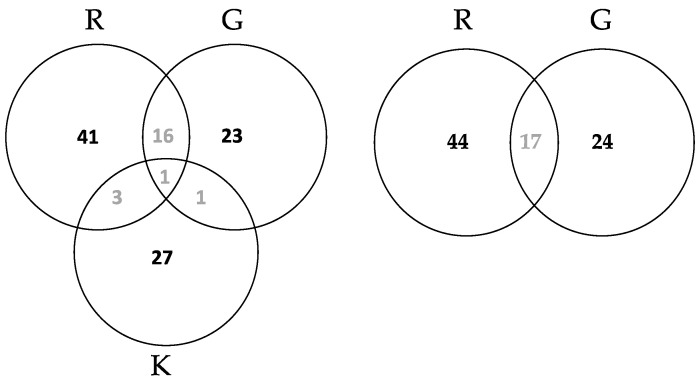
Number of unique and common OTUs isolated from leaf and root samples from Roosky, Glasnevin, and Kinsealy. Leaf endophyte Roosky = R, Glasnevin = G, and root endophytes Kinsealy = K. Root endophytes were only isolated from Kinsealy.

Altogether, 410 isolates were used for DNA barcoding; 310 were leaf endophytes and 100 were root endophytes and we successfully amplified and sequenced 390 isolates (20 remained unidentified). No seed endophytes were obtained by culturing. The nrITS region was found to be the most efficient for fungal identification compared to nrLSU and *tef*, so most samples were identified using nrITS (GenBank accessions numbers MZ509281—MZ5099373; and MZ506890—MZ506894). From these 390 sequences, we obtained 112 different OTUs. The frequency of each of the OTUs at each of the sites are given in Supplementary Table S4 and summarised in the Venn diagrams (Figure 1).

The most abundant leaf OTUs were *Boeremia exigua*, *Cladosporium* sp., *Diaporthe* spp., *Epicoccum nigrum*, *Fusarium* sp., *Mycosphaerella* sp., and *Phoma* spp. Some OTUs were only recorded in root tissues (*Ilyonectria* sp., *Nectria* sp., and *Neonectria* sp.). *Boeremia exigua* and *Mycosphaerella coacervata* were frequently isolated from Roosky material (58 and 28 of the isolates, respectively). Moderate frequencies were recorded for *Cladosporium* sp., *Dactylonectria macrodidyma*, *Fusarium laterium*, and *Phoma* sp. (Supplementary Table S4).

We found 374 isolates belonging to Ascomycota and 16 to Basidiomycota. The samples belonged to 10 different taxonomic classes (Supplementary Table S5), among which the most abundant classes were Dothideomycetes and Sordariomycetes, having 195 and 162 isolates, respectively (40, 47 OTUs, respectively). The number of isolates and OTUs in other classes ranged from 1 to 7. Most of the isolated endophytes belonged to the subdivision Pezizomycotina (372), a moderate number to Agaricomycotina (13) and very few to Pucciniomycotina (1), Saccharomycotina (2), and Ustilaginomycotina (2). A total of 37 families (Supplementary Table S5) were represented, within which Didymellaceae and Nectriaceae were most frequent (104 and 89 isolates, respectively). Isolates belonging to Cladosporiaceae, Diaportheceae, and Mycosphaerellaceae were also moderately common; 21, 20, and 45 isolates, respectively. 

Leaf endophytes isolated from the provenance trial of *Fraxinus excelsior* at Roosky were tabulated according to country of origin (Supplementary Table S6). The OTU frequency from sampled leaf tissue for each provenance is shown. The total number of taxa per provenance ranged from 8 (Czech Republic and the Netherlands) to 19 (Ireland). The genera *Boeremia*, *Diaporthe*, *Fusarium, Mycosphaerella*, and *Phoma* were found in most provenances.

#### 3.1.1. Endophytes in Other *Fraxinus* Species

A comparison was made of leaf endophytic taxa obtained from *Fraxinus excelsior* and other *Fraxinus* species (*F. americana*, *F. angustifolia*, *F. dipetala*, *F. glabra*, *F. mandshurica*, *F. numidica*, *F. ornus*, *F. pennsylvanica*, *F. potamophila*, *F. pubinervis*, *F. texensis*, *F. xanthoxyloides*) from Glasnevin (Supplementary Table S4). A total of 69 leaf endophyte taxa (from n = 229) were obtained from *F. excelsior* and a total of 36 leaf endophyte taxa for other *Fraxinus taxa* (from n = 54). Sixteen taxa were shared between *F. excelsior* and the other *Fraxinus taxa* group (*Acremonium* sp., *Aureobasidium pullulans*, *Aureobasidium* sp., *Boeremia exigua*, *Cladosporium* sp., *Diaporthe rudis*, *Diaporthe viticola*, *Fusarium avenaceum*, *F. lateritium*, *F. oxysporum*, *F. proliferatum*, *Fusarium* sp., *Gloniopsis calami*, *Mycosphaerella coacervata*, *Parengyodontium album*, and *Phoma* sp.). Some taxa were only isolated from *Fraxinus excelsior* (53 taxa) and some only from the other *Fraxinus* species (20 taxa) (Supplementary Table S4).

#### 3.1.2. OTU Retrieval from Different Leaf Tissue Types and Roots in Fraxinus

The frequencies of OTUs retrieved from each type of healthy tissue are shown in the heat-map in Supplementary Figure S1. *Boeremia* sp., *Diaporthe* sp., *Fusarium* sp., *Mycosphaerella* sp., and *Phoma* sp. were frequently isolated from healthy rachis (H.R) material. The total number of isolates (53) and taxa (11) were also the highest for the healthy rachis material. Healthy leaf tissue (H.L) also provided a high number of isolates and taxa (43, 11, respectively). *Boeremia* sp., *Cladosporium* sp., *Diaporthe* sp., *Leptosphaerilina* sp., *Mycosphaerella* sp., *Parengyodontium* sp., and *Septoria* sp. were very frequent in the H.L. The highest number of isolates and OTUs from diseased tissues (Supplementary Figure S1) was obtained from leaf tissue (D.L) (34 isolates, 16 OTUs, respectively) and midrib (D.M) material (33 isolates, 18 OTUs) with *Boeremia* sp., *Fusarium* sp., *Phaeosphaeria* sp., *Mycosphaerella* sp., *Phoma* sp., and *Septoria cucubali* being particularly common. The total number of different taxa obtained from all diseased tissues was 18 compared to 33 for healthy leaf tissue. Four of the families detected were only found in diseased samples; Psathyrellaceae (Agaricales) and Bulleribasidiaceae (Tremellales) were only found from Roosky and Hysteriaceae (Hysteriales) and Diatrypaceae (Xylariales) were only found from Glasnevin.

Root endophytes were only obtained from Kinsealy (Table 1; Supplementary Table S4). Most taxa were specific to root tissues and not found in leaf material from the other sites. *Cadophora* sp., *Cladosporium cladosporioides*, *Epicoccum nigrum*, *Fusarium culmorum*, and *F. oxysporum* were found in both leaf and root material. Root endophytes were split into four classes, six orders, and ten families; three of which were unique to roots, namely Bionectriaceae, Ceratostomataceae, and Clavicipitaceae. Basidiomycetes were only detected in the roots. In contrast, 26 families were unique to leaves, namely Bulleribasidiaceae, Chaetosphaeriaceae, Corticiaceae, Cystobasidiaceae, Debaryomycetaceae, Dermataceae, Diaporthaceae, Diatrypaceae, Didymosphaeriaceae, Dothioraceae, Filobasidiaceae, Glomerallaceae, Herpotrichiellaceae, Hydnaceae, Hysteriaceae, Lasiosphaeriaceae, Meruliaceae, Mycosphaerellaceae, Phaeosphaeriaceae, Pleosporaceae, Psathyrellaceae, Pyronemataceae, Sarocladiaceae, Sclerotiniaceae, Xylariaceae, and Ustilaginaceae (Supplementary Tables S4 and S5).

### 3.2. Community Analysis of Samples Using Direct Amplicon High Throughput Sequencing (HTS)

#### 3.2.1. Diversity of the Ash Microbiome by HTS

A total of 324 OTUs was obtained from the 70 leaf and seed samples from the nrITS amplicon HTS. From these, 212 were assigned to the kingdom Fungi and 3 to kingdom Chromista. Other OTUs were assigned to kingdom Plantae and 16 reads were assigned to unidentified fungal isolates. The Plantae sequences were a mixture of *Fraxinus* (the host species) and various algae. Some of these are likely to be epiphytic on the surface of the ash leaves.

Samples used for HTS were selected to cover a wide range of provenances, tissue types, and *Fraxinus* taxa (Supplementary Table S2). Alpha diversity of the samples from the HTS data were assessed by rarefaction curves and rank abundance curves (Supplementary Figures S2 and S3). The rarefaction curves (Supplementary Figure S2) for most samples started to level off as early as 7309 reads and had levelled off reasonably well by the maximum 43,804 reads per sample (our budget was limited to 50,000 raw tags (=43,804 cleaned reads). The samples from F3 (France, Lake Annecy) and R1 (Ireland, Enniskillen) showed the most OTUs, and S1 seed DNA (France, St Paul De Salers) and S5 (Ireland, Enniskillen) the fewest. The species richness of F3 was strikingly higher than all other samples. The rank abundance curves (Supplementary Figure S3) showed that approximately 20 OTUs were relatively abundant in most samples, but most species were rare (small number of reads). 

**Figure 2:**
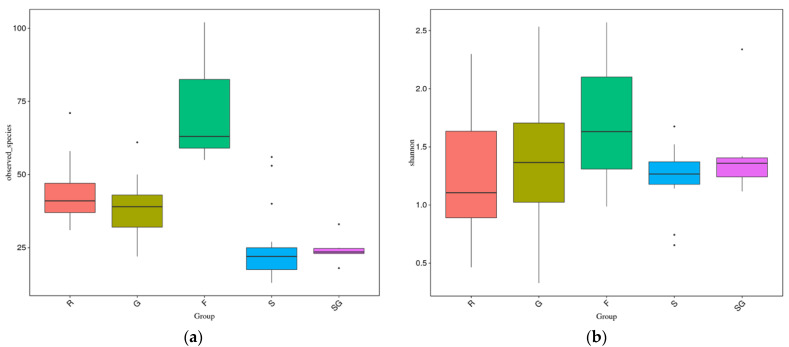
Box plots of fungal species richness (**a**) and Shannon indices (**b**) in the five different HTS ITS amplicon groups. R = Roosky leaves, G = Glasnevin leaves, F = France leaves; S = Roosky seeds, SG = Glasnevin seeds. All pairwise species richness comparisons were significantly different (*p* < 0.05) with Tukey tests except R-G, SG-G, and SG-S; and for Wilcoxon tests, all were significantly different (*p* < 0.05) except for R-G and SG-S. For the Shannon index values, none of the pairwise comparisons were significantly different with either the Tukey or Wilcoxon tests.

The highest diversity in terms of species richness and Shannon’s index was found for the French leaf group (Figure 2). Relatively high species richness (=OTU number) was also found for the Roosky and Glasnevin leaf groups. Lowest species richness was found in the two seed groups from Roosky (S) and Glasnevin (SG). However, the seed groups had relatively higher Shannon index values (comparable to leaves from Roosky and Glasnevin). For species richness values, all pairwise comparisons are significantly different (*p* < 0.05) with Tukey tests except R-G, SG-G and S-SG; and for the Wilcoxon test, all were significantly different (*p* < 0.05) except for R-G and S-SG. For the Shannon index, none of the pairwise comparisons were significantly different with either the Tukey or Wilcoxon tests.

#### 3.2.2. Taxonomic Composition of HTS Data

The results for the 70 seed and leaf DNA samples for nrITS amplicon sequencing by HTS were classified by taxonomic rank. Fungi were detected in four phyla (Ascomycota, Basidiomycota, Zygomycota, and Chyridiomycota). Ascomycota and Basidiomycota dominated the leaf samples, but Zygomycota were relatively abundant in seeds from Glasnevin, and Chytridiomycota relatively abundant in seeds from Roosky (Supplementary Figure S4). The abundance of fungal classes varied according to geographic source (Roosky, Glasnevin, France) or tissue type (seed or leaf). Among them, Sordariomycetes and Chytridiomycetes were relatively more abundant in seed samples from Roosky, and incertae sedis Zygomycota was high in seed samples from Glasnevin. Leotiomycetes were relatively abundant in Roosky leaf samples whereas Lecanoromycetes and Microbotryomycetes were relatively abundant for Glasnevin samples. Many taxonomic classes were relatively abundant in the French samples, but rarer in the other samples such as Agaricomycetes, Taphrinomycetes, Dothideomycetes, Eurotiomycetes, Tremellomycetes, and Cystobasidiomycetes (Supplementary Figure S4 and Table S7).

The samples are grouped into 35 families (Supplementary Figure S4). Dominant groups of families and genera differed for site and tissue type. For example, 17 families were dominant in French leaf material but rare in other samples. Diaporthaceae, Nectriaceae, Ceratostomataceae, and an *incertae sedis* Pleoporid were abundant in seed from Roosky, and Mucoraceae was abundant in seed from Glasnevin. Thirty-five genera were found in all 70 DNA samples by HTS. Higher abundance of 17 genera was found in leaf samples from France including *Alternaria* sp., *Ascochyta* sp., *Bensingtonia* sp., *Botryosphaeria* sp., *Bullera* sp., Cryptococcus sp., *Fomitopsis* sp., *Leptosphaeria* sp., *Peniophora* sp., *Phaeosphaeria* sp., *Phomopsis* sp., *Sterilitziana* sp., *Trichomerium* sp., *Trimmatostroma* sp., *Veturia* sp., *Wojnowicia* sp., and *Zymoseptoria* sp. In Glasnevin leaves, *Microstroma* sp. and *Rhodotorula* sp. were dominant and in leaves from Roosky, *Acicuseptoria* sp., *Cadophora* sp., *Hannaella* sp., *Phyllactinia* sp., and *Septoriella* sp. had high scores. Seed DNA from Roosky showed five genera (*Diapothe* sp., *Harzia* sp., *Neofusicoccum* sp., *Paraconiothyrium* sp., and *Volutella* sp.) of relatively high abundance and two genera (*Mucor* sp. and *Talaromyces* sp.) of high abundance from Glasnevin (Supplementary Figure S4). 

#### 3.2.3. Community Structure and Differences of HTS Data

**Figure 3:**
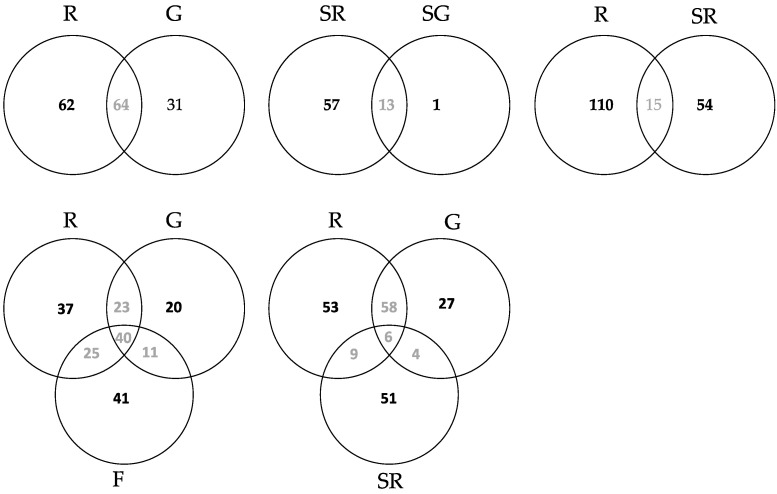
Total OTU number within and shared among groups using high throughput Illumina sequencing. R = Roosky leaves, G = Glasnevin leaves, F = France leaves; SR = Seeds Roosky, SG = Seeds Glasnevin.

The Venn diagrams in Figure 3 show the distribution of OTUs among groups (the unique OTUs in each group and shared OTUs among groups under differing combinations). The R–G–F Venn diagram comparison (lower left) shows the leaf endophyte data for Roosky, Glasnevin, and France. Each site had a large number of unique leaf OTUs (37, 20, and 41, respectively), but also a high number of shared OTUs. Forty leaf OTUs were shared among all locations. Sixty-four leaf OTUs were shared between Roosky and Glasnevin (upper left), 65 between Roosky and France, and 51 between Glasnevin and France.

Comparisons of OTU richness in seeds are also shown in the Venn diagrams in Figure 3. Roosky had a high number of unique seed endophytes (57) compared to one unique seed endophyte from Glasnevin (top right). However, the two groups also shared 13 core seed OTUs. A total of 15 endophyte OTUs were shared among Roosky leaves and Roosky seeds (bottom right), but these groups had more unique (110, 54) than shared OTUs. Seeds from Roosky shared a total of 6 OTUs with leaves from Roosky and Glasnevin combined (middle bottom).

**Figure 4:**
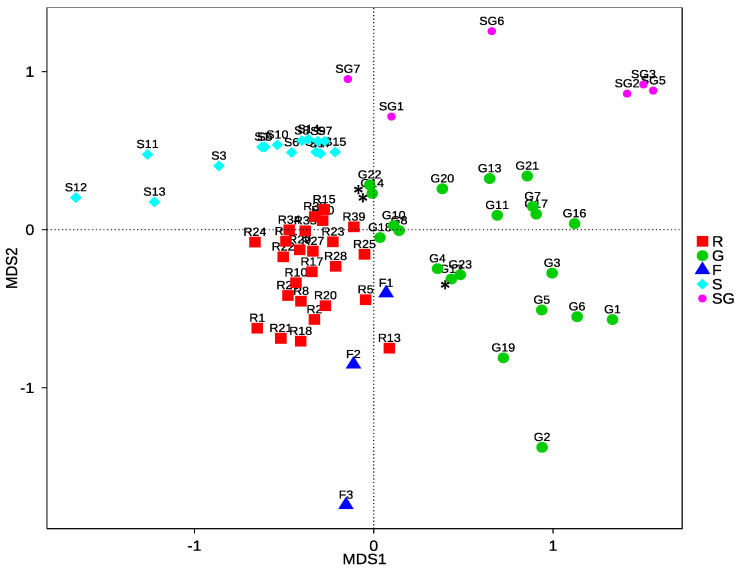
NMDS plot of leaf and seed HTS samples from Roosky, Glasnevin, and France. Legends on the right corresponds to R = leaves from Roosky, G = leaves from Glasnevin, F = leaves from France, S = Seed Roosky, and SG = Seed Glasnevin. Sample number is provided. Stress = 0.180. All samples are from *F. excelsior* except from the mixed *Fraxinus* taxon sample from G. For G, samples G12, G14, and G22 (highlighted with *) are from *Fraxinus excelsior* and all other G samples are from different *Fraxinus* species/taxa (as outlined in Table S2).

Figure S5 shows a PCoA with unweighted unifrac distances. It groups samples into rough clusters corresponding to either their geography (Roosky, Glasnevin, France) or tissue type (leaf or seed). A similar pattern can be seen in the NMDS plot (Figure 4). The Glasnevin sample is a diverse set of *Fraxinus* species in comparison to the Roosky and French samples that are only *F. excelsior*. The French samples grouped more closely with Roosky samples than in the Glasnevin samples even though Roosky and Glasnevin are in Ireland. This indicates that plant host species identity has a strong influence on fungal community composition. F3 was the French sample with much higher species richness than the others. The *F. excelsior* samples in the mixed *Fraxinus* species group from Glasnevin also grouped closely to the Roosky and French *F. excelsior* groups in the NMDS (Figure 4; asterisks). The endophyte communities of seeds from Glasnevin and Roosky were also relatively distinct.

The highest beta diversity levels (Supplementary Figure S6) were found between seed and leaf groups and between the France and Ireland groups. All comparisons of beta diversity with weighted unifrac distances were significantly different with Tukey HSD at *p* < 0.05, except for G-F, SG-G, and S-R (in Wilcoxon tests all were significantly different except for G-F, F-SG, SG-G, and S-R). With unweighted unifrac distances, only R-G, S-G, S-R, and SG-R were significantly different with Tukey HSD (in Wilcoxon tests, R-G, S-G, S-R, and SG-R, F-R, and SG-G were significantly different at *p* < 0.05 level).

The leaf OTUs detected by the culture independent approach were divided into four phyla, 16 classes, 32 orders, and 42 families and detected many fungal taxa not detected by the culture dependent method, belonging to (a) three phyla (Chytridiomycota, Zygomycota and Oomycota); (b) eight classes (Agaricostilbomycetes, Chytridiomycetes, Exobasidiomycetes, Lecanoromycetes, Microbotryomycetes, Mucoromycetes, Oomycetes, and Taphrinomycetes); (c) fifteen orders (Agaricostilboles, Botryosphaeriales, Cystofilobasidiales, Erysiphales, Entylomatales, Lecanorales, Melanosporales, Micostromatales, Myriangles, Russulales, Sporidiobolales, Verrucariales, Venturiales, Taphrinales and Teloschistales); and (d) thirty families (Agaricostilbaceae, Ascobolaceae, Botryosphaeriaceae, Bulleraceae, Capnodiaceae, Ceratostomataceae, Chaetothyriaceae, Cystofilobasidiaceae, Elsinoaceae, Entylomataceae, Erysiphaceae, Fomitopsidaceae, Kondoaceae, Leptosphaeriaceae, Microstomataceae, Montagnulaceae, Mucoraceae, Peniophoraceae, Pythiaceae, Ramalinaceae, Rhizopodaceae, Sordariaceae, Sporidiobolaceae, Taphrinaceae, Teloschistaceae, Tremellaceae, Trichocomaceae, Venturiaceae, Verrucariaceae, and Vibrisseaceae) (Supplementary Table S7). 

In addition, a total of 11 classes were detected in leaves with direct amplicon sequencing compared to 9 in the culture dependent sample (Supplementary Table S7). However, the culture dependent method detected many taxa not detected by the culture independent method for leaves including two classes (Saccharomycetes and Ustilaginomycetes), 10 orders (Agaricales, Cantharellales, Chaetosphaeriales, Corticiales, Cystobasidiales, Filobasidiales, Hysteriales, Saccharomycetales, Ustilaginales, and Xylariales), and 26 families (Supplementary Table S7). 

Several core taxa could be identified in the leaf samples present in both Roosky and Glasnevin using the HTS approach. These differed markedly from the core leaf taxa identified by the culture dependent method and Sanger sequencing (Table 1). Core taxa were defined here as taxa that that were always present. None of these were shared between the culture dependent and culture independent methods. 

## 4. Discussion

### 4.1. Culture Dependent Fungal Diversity and Community Composition

#### 4.1.1. Diversity and Taxonomy of Endophytes from the Culture Dependent Sample

Using the culture dependent approach, we identified 141 OTUs in total from the leaf and root endophyte culture samples (Supplementary Table S4). Cultured leaf endophyte isolates from the Roosky provenance trial of *Fraxinus excelsior* and the mixed *Fraxinus* species sample from Glasnevin belonged to two divisions (Ascomycota and Basidiomycota) and 7 and 9 classes, respectively (Basidiomycota: Agaricomycetes, Cystobasidiomycetes, Tremellomycetes; Ascomycota: Dothideomycetes, Eurotiomycetes, Leotiomycetes, Pezizomycetes, Saccharomycetes, Sordariomycetes, and Ustilaginomycetes). SubdivisionPezizomycota were the most common and this was consistent with Murphy et al. [56] for barley using a culture dependent approach. The root fungal community was found to be highly different to the leaf communities and were split into ten families; three of which were unique to roots. In contrast 26 families were unique to leaves. 

Other studies on the culture dependent ash mycobiome by Bakys et al. [39,44,48,57] have found a combined study total of 133 taxa, 63 families, and 17 orders belonging to two divisions (Ascomycota and Basidiomycota) and three subdivisions (Agaricomycotina, Mucoromycotina, Pezizomycotina). We also found Ascomycotina and Basidiomycotina, from our cultured leaf and root endophytes, but detected more subdivisions (five compared to three) including 119 OTUs divided among 35 families, 23 orders, and 11 classes (Supplementary Table S5). 

We found several distinct taxa from our cultured leaf and root endophytes that were not found in these previous studies including 16 unique families: Bionectriaceae, Bulleribasidiaceae, Ceratomataceae, Chaetosphaeriaceae, Clavicipitaceae, Corticiaceae, Cystobasidiaceae, Debaryomycetaceae, Filobasidiaceae, Herpotrichiellaceae, Hysteriaceae, Lasiosphaeriaceae, Meruliaceae, Psathyrellaceae, Pyronemataceae, and Ustilaginaceae. 

Kowalski and Lukomska [58] studied endophytes from three types of diseased tissues, namely dying top shoots, local canker, and dead roots, using a culture dependent approach. They found no common genera among roots and shoots. Three genera were found from dead roots, namely *Pezicula radicicola* (=*Cryptosporiopsis radicicola), Ilyonectria destructans* (=*Cylindrocarpon destructans*), and *Phialocephala* sp. However, we did not find these genera in our root samples. Kowalski and Lukomska [58] also found *Cladosporium cladosporioides*, *Cytospora populina* (=*Cytospora ambiens*), *Fusarium lateritium*, *Gloeosporidiella turgida*, *Hymenoscyphus* sp., and two species of *Phomopsis* sp. We found some of these, namely *Cladosporium cladosporioides* (healthy leaf and root tissue in Roosky), *Fusarium lateritium* (healthy and diseased leaf tissue in Roosky and Glasnevin), and *Phomopsis* sp. (healthy root tissue from Kinsealy) (Supplementary Table S4). 

#### 4.1.2. Community Differences Detected from the Culture Dependent Approach

The leaf samples from Roosky and Glasnevin had more unique OTUs than shared OTUs (Figure 1). This difference can be explained by host species effects (as the Glasnevin sample was from a range of *Fraxinus* taxa compared to the single *Fraxinus excelsior* species at Roosky). It can also be explained by habitat and ecological differences among sites. Glasnevin is a botanic garden and Roosky is a provenance trial plantation. Seventeen OTUs were shared among these sets and can be considered core endophytes. There was also a high proportion of unique endophytes in the roots from Kinsealy (28), with only five shared with the leaf samples in total and only one OTU (*Fusarium oxysporum*) shared among Roosky (leaf), Glasnevin (leaf), and Kinsealy root (Figure 1). It is not known if the five OTUs (*Cadophora* sp., *Cladosporium cladospioroides*, *Epicoccum nigrum*, *Fusarium culmorum*, and *Fusarium oxysporum*) shared by leaves and roots are transmitted vertically by seed, but it is a hypothesis worth investigating further.

Therefore, the culturable endophyte communities from differing sites, tissue type (roots and shoots), and tissue health (diseased and healthy) have been demonstrated to be highly different from each other. The culturable community are the sample that can be used for further experimentation and potential biocontrol of ash dieback disease. Our sampling has ensured that we captured maximal diversity for one timepoint of sampling. 

### 4.2. Culture Independent Fungal Diversity and Community Composition 

#### 4.2.1. Diversity in the Culture Independent Sample 

The highest alpha diversity in terms of species richness and Shannon’s index was found for the French leaf group (Figure 2). Relatively high species richness was also found for the Roosky and Glasnevin leaf groups. Lowest species richness was found in the two seed groups from Roosky (S) and Glasnevin (SG). No seed was sampled from France. However, the seed groups had relatively higher Shannon index values (comparable to leaves from Roosky and Glasnevin). Thus, they are much more species poor but have comparable evenness. It is not known why the French samples had the highest richness despite being the smallest sample, however, it could be due to the isolated island status of Ireland, which hosts lower animal and plant diversity [59] and might also be expected to host lower fungal diversity. The French population was also from a natural forest in the Alps compared to a plantation in Roosky and botanic garden in Glasnevin. 

#### 4.2.2. Community Differences 

The fungal communities of each geographical location (Roosky, Glasnevin, France) and plant tissue type (leaf vs. seed) differed considerably. The highest beta diversity levels were found between the seed and leaf groups and between the France and Ireland groups. The NMDS (Figure 4) and PCoA (Figure S5) grouped samples into rough clusters corresponding to either their geography (Roosky, Glasnevin, France) or tissue type (leaf or seed). The Glasnevin sample is a diverse set of *Fraxinus* species in comparison to the Roosky and French samples that are only *F. excelsior*, which might partly explain their difference. The French leaf samples grouped more closely with the Roosky samples than the Glasnevin samples (despite the close geographical proximity of Roosky and Glasnevin). The *F. excelsior* samples in the Glasnevin botanic garden sample also grouped closely to the other *F. excelsior* samples in the NMDS. Thus, there is a strong influence of *Fraxinus* species on fungal community composition. 

The Venn diagrams (Figure 3), PCoA (Supplementary Figure S5) and NMDS (Figure 4) also showed that the endophyte communities of seeds were distinct from those of leaves. In addition, the seed endophyte community from Glasnevin and Roosky were also relatively distinct. *Aspergillus niger, A. penicillioides, Harzia acremonioides, Mucor abundans, Paracamarosporium hawaiiense* (*=Paraconiothyrium hawaiiense*)*, Penicillium brevicompactum,* and *Volutella ciliata* were present in seeds from both sites and can be considered core endophytes of ash seeds (Supplementary Table S8). *Diplodia mutila* (=*Botryosphaeria stevensii*) is a known pathogen of ash [44] and was commonly present in leaf samples from Glasnevin and Roosky and seed from Roosky. Likewise, *Sordaria fimicola* was commonly present in leaf samples from Roosky and seed samples from Glasnevin (Supplementary Table S8). 

Some of the seed endophytes have also been reported in other plants including ash. *Harzia acremonioides* was reported from syptomatic ash petiole in northwestern Spain [60]; *Diplodia mutila* (=*Botryosphaeria stevensii*) has also been reported from twigs of *Vitis vinifera* [61]; *Volutella ciliata* has been reported as a culturable endophyte from roots of *Pinguicula vulgaris* [62]; *Paraconiothyrium brasiliense* was reported for the first time from Chinese maple leaves [63]; *Talaromyces minioluteus* was isolated from *Silybum marianum* and produces biological active compounds [64]; and *Aspergillus* sp. and *Penicillium* sp. were reported as endophytes from *Taxus globosa* [65]. Hayatgheibi [66] isolated *Lophodermium pinastri* as an endophyte from ash seeds and *Paracucurbitaria corni* (=*Pyrenochaeta corni*) was noted as an endophyte of ash seeds [67]. In the present study, we did not isolate these endophytes from seeds. 

### 4.3. Comparison of OTUs Obtained from High Throughput Amplicon Sequencing (HTS) and Sanger Sequencing

Very large differences were found in OTU diversity and community composition recorded by the culture dependent) and culture independent approach (direct HTS sequencing of plant material), despite the fact that the same nrITS DNA barcoding region was used in each. The taxonomic breadth of fungal OTUs was much higher in the culture independent approach. Two subdivisions were obtained from the culture dependent sequencing approach (Agaricomycotina and Pezizomycotina), but fungi were detected from five subdivisions using the culture independent HTS sequencing approach, namely Agaricomycotina, Pezizomycotina, Pucciniomycotina, Taphrinomycotina, and Ustilaginomycotina (Supplementary Table S7).

It was particularly noteworthy that only two OTUs were shared between the two approaches (*Alternaria* sp. and *Phaeosphaeria* sp.). Core OTUs found at both Irish sites (Roosky and Glasnevin) are listed in Table 1. None of these were shared between the culture dependent and culture independent methods. The nrITS region for fungal identification was the same, so the results cannot be explained by database coverage. However, the primers differed to amplify the nrITS region. Furthermore, it is known that many fungi such as those in the Glomerales are not directly culturable. However, the culturable fungi would be expected to be detected by the direct HTS of plant material as they are present. Perhaps they are rarer than expected and are not detected because of competitive template processes in the PCR. Therefore, these data from the two approaches need to be combined to better estimate core OTU diversity in differing environments and among differing species.

Other studies on the ash mycobiome have been conducted using the HTS method, but only a small subset of taxa was shared with our analysis. *Phyllactinia* sp. was found by Cross et al. [45] to be positively correlated with *Hymenoscyphus fraxineus* and *Taphrina* sp.; *Tilletiopsis* sp. endophytes were negatively correlated. We also found *Phyllactinia fraxini, Tilletiopsis washingtonensis,* and five species of *Taphrina* sp. from our healthy leaf samples. However, we did not detect *Hymenoscyphus fraxineus* from those leaves. A high number of reads (14,901) of *Phyllactinia fraxini* suggest that it is a core mycobiome component of ash leaves. Cleary et al. [47] found *Mycosphaerella* sp., *Cladosporium* sp., and *Phoma* sp. commonly present in asymptomatic leaves of *Fraxinus mandshurica*. Our study supports this finding because we also found *Mycosphaerella* sp., *Cladosporium* sp., and *Phoma* sp. as commonly present in healthy tissues but rarely in diseased tissue (one isolate). These three taxa were cultured but not detected with the HTS method. Higher numbers of isolates were also found for *Phoma* sp. in healthy and diseased tissue sampled from the Roosky site. Species boundaries in *Phoma* and many other fungal genera in our study are unclear [68], but despite this taxonomic uncertainty, it is clear that *Phoma* is commonly associated with *Fraxinus excelsior*. 

Schlegel et al. [40] compared culture dependent and independent methods (HTS Illumina data) in ash. They found that the most abundant OTUs for a diseased geographical area were *Mycosphaerella* sp., two *Cladosporium* spp., *Preussia minima*, and one *Venturia fraxini* genotype. For the symptomless area, the most abundant OTUs were *Paraconiothyrium* sp., *Colletotrichum godetiae*, and another *Mycosphaerella* sp. We have only sampled healthy leaf tissues and seeds for the culture independent method and found two species of *Paraconiothyrium*, but not from leaf samples from Roosky and Glasnevin (Table 1). Cleary et al. [47] also found *Paraconiothyrium* sp. from leaves of *Fraxinus mandshurica* in East Russia. We isolated *Paraconiothyrium* sp. by the culture dependent method from leaf samples of *Fraxinus excelsior* at the Roosky site (Supplementary Table S4). According to Schlegel et al. [40], *Paraconiothyrium* sp. has strong antibiotic compounds that can inhibit ascospore germination and mycelial growth [48] of *H. fraxineus*. 

Agostinelli [46] studied the mycobiome of *Fraxinus excelsior* from leaf, bark., and xylem using a culture independent HTS method and commonly retrieved *Aureobasidium pullulans*, *Alternaria* sp., *Phomopsis* sp., and *Trichoderma* sp. In the present study, we found *Alternaria* sp. using both methods, but only detected *Aureobasidium pullulans* (from leaf samples from Roosky and Glasnevin), *Phomopsis* sp. (leaf sample at Glasnevin), and *Trichoderma* sp. (root sample, Kinsealy) by the culture dependent method. 

## 5. Conclusions

This study revealed extensive endophyte diversity in *Fraxinus excelsior* and other *Fraxinus* taxa. Only a few taxa were shared between the two approaches of sampling endophytic diversity and the two lists need to be combined to fully represent the diversity present. The ash dieback pathogen, *Hymenoscyphus fraxineus*, was not detected by either approach in any of the samples even though it was known to exist in the French samples and in Roosky. It is also clear that different sampling localities and different tissues (especially roots, leaves, and seeds) support largely different communities. The culturable endophytes are of most interest for practical application as they represent the taxa that are now immediately available for testing the role of the microbiome on the biotic or abiotic stress resistance in ash [6,53] and developing microbiome related management strategies or treatments for diseases of ash such as *H. fraxineus*.

## Figures and Tables

**Table 1:** Core taxa found at both Roosky and Glasnevin obtained from Sanger sequencing of the isolates or by HTS of leaf samples using nrITS DNA.

Sanger Culture Dependent	HTS Culture Independent
*Acremonium* sp.	*Alternaria infectoria* *
*Alternaria humuli* *	*Bacidina flavoleprosa*
*Aureobasidium pullulans*	*Cadophora orchidicola*
*Aureobasidium* sp.	*Diplodia mutila*
*Boeremia exigua*	*Microstroma bacarum*
*Cladosporium* sp.	*Nematostoma parasiticum*
*Diaporthe rudis*	*Paraleptosphaeria rumicis*
*Diaporthe viticola*	*Peniophora cinerea*
*Fusarium lateritium*	*Phaeosphaeria caricicola* *
*Fusarium oxysporum*	*Phyllactinia fraxini*
*Fusarium proliferatum*	*Pseudomicrostroma juglandis*
*Mycosphaerella coacervata*	*Sclerostagonospora opuntiae*
*Paraloratospora gahniae* *	*Septoriella phragmitis*
*Parengyodontium album*	*Septoriella vagans* *
*Phaeosphaeria pontiformis* *	*Strelitziana eucalypti*
*Phoma* sp.	*Taphrina antarctica*
*Phomopsis velata*	*Trimmatostroma cordae*
*Pyronema domesticum*	*Xanthoria parietina*

* indicates common genera detected by both approaches from the Roosky site that are not present in the Glasnevin site.

## Data Availability

DNA sequences are deposited in GenBank under accession numbers MZ509281—MZ5099373; and MZ506890—MZ506894.

## Supplementary Materials

The following are available online at https://www.mdpi.com/article/10.3390/jof7070565/s1, Figure S1: Heat-map for mean frequency of OTUs in different leaf tissue types; Figure S2: Rarefaction curves for all HTS samples; Figure S3: Rank abundance curves for all samples; Figure S4: OTU abundance heat map categorised into phyla, classes, families, or genera of the 70 samples screened with HTS of nrITS; Figure S5: PCoA with unweighted unifrac distances; Figure S6: Heat-map for beta diversity. Table S1: Ash leaflets, seed, and roots collected for fungal isolations, DNA extraction, and HTS; Table S2: Samples selected for HTS Ilumina analysis and their quantity of total DNA; Table S3: Primer information for each DNA barcoding locus used (nrITS, *tef*, and LSU). Table S4: Frequencies for each of the OTUs at the study sites; Table S5: Total number of isolates and OTUs within each class and family from all leaf and root endophytes; Table S6: OTU frequencies obtained from all sampled leaf tissues from all provenances in the Roosky provenance trial for *Fraxinus excelsior*; Table S7: OTUs assigned to respective taxonomic level with total numbers of OTU obtained from seeds and leaves by HTS in the culture independent assessments; Table S8: Dominant fungal endophytes identified from seed taken from two sites, Roosky and Glasnevin, using the HTS of plant material. OTUs highlighted in bold were shared among sites and considered core.

## References

[1] Vorholt J.A. (2012). Microbial life in the phyllosphere. Nat. Rev. Microbiol..

[2] Newton A.C., Fitt B.D.L., Atkins S.D., Walters D.R., Daniell T.J. (2010). Pathogenesis, parasitism and mutualism in the trophic space of microbe-plant interactions. Trends Microbiol..

[3] Knief C. (2014). Analysis of plant microbe interactions in the era of next generation sequencing technologies. Front. Plant Sci..

[4] McEvoy A., O’Regan F., Fleming C.C., Moreland B.P., Pollock J.A., McGuinness B.W., Hodkinson T.R. (2016). Bleeding canker of horse chestnut (*Aesculus hippocastanum*) in Ireland: Incidence, severity and characterization using DNA sequences and real-time PCR. Plant Pathol..

[5] Hodkinson T.R., Doohan F.M., Saunders M.J., Murphy B.R. (2019). Endophytes for a Growing World.

[6] Lahiri A., Douglas G.C., Murphy B.R., Hodkinson T.R., Hodkinson T.R., Doohan F.M., Saunders M.J., Murphy B.R. (2019). In vitro methods for plant-microbe interaction and biocontrol studies in European ash (*Fraxinus excelsior* L.). Endophytes for a Growing World.

[7] Strobel G.A., Daisy B., Castillo U., Harper J. (2004). Natural products from endophytic microorganisms. J. Nat. Prod..

[8] Sudhakar T., Dash S., Rao R., Srinivasan R., Zacharia S., Atmanand M., Subramaniam B., Nayak S. (2013). Do endophytic fungi possess pathway genes for plant secondary metabolites?. Curr. Sci..

[9] Aly A.H., Debbab A., Proksch P. (2011). Fifty years of drug discovery from fungi. Fungal Divers..

[10] Crous P.W., Braun U., Schubert K., Groenewald J.Z. (2007). Delimiting *Cladosporium* from morphologically similar genera. Stud. Mycol..

[11] KoKo T.W., Stephenson S.L., Bahkali A.H., Hyde K.D. (2011). From morphology to molecular biology: Can we use sequence data to identify fungal endophytes?. Fungal Divers..

[12] Olson Å., Stenlid J. (2002). Pathogenic fungal species hybrids infecting plants. Microbes Infect..

[13] Kohn L.M. (2005). Mechanism of fungal speciation. Ann. Rev. Phytopathol..

[14] Foltz M.J., Perez K.E., Volk T.J. (2013). Molecular phylogeny and morphology reveal three new species of Cantharellus within 20 m of one another in western Wisconsin, USA. Mycologia.

[15] Giraud T., Refrégier G., Le Gac M., de Vienne D.M., Hood M.E. (2008). Speciation in fungi. Fungal Genet. Biol..

[16] Moore D., Robson G.D., Trinci A.P.J. (2011). 21st Century Guidebook to Fungi.

[17] White T.J., Bruns T., Lee S., Taylor J., Innis M.A., Gelfand D.H., Sninsky J.J., White T.J. (1990). Amplification and direct sequencing of fungal ribosomial RNA genes for phyologenetics. PCR Protocols: A Guide to Methods and Applications.

[18] Gardes M., Bruns T.D. (1993). ITS primers with enhanced specificity of basidiomycetes: Application to the identification of mycorrhizae and rusts. Mol. Ecol..

[19] Schoch C.L., Seifert K.A., Huhndorfc S., Robertd V., Spougea J.L., Levesqueb C.A., Chen W. (2012). Fungal Barcoding Consortium. Nuclear ribosomal internal transcribed spacer (ITS) region as a universal DNA barcode marker for Fungi. Proc. Natl. Acad. Sci. USA.

[20] Begerow D., Nilsson H., Unterseher M., Maier W. (2010). Current state and perspectives of fungal DNA barcoding and rapid identification procedures. Appl. Microbiol. Biotechnol..

[21] Seifert K.A. (2009). Progress towards DNA barcoding of fungi. Mol. Ecol. Res..

[22] Reeb V., Lutzoni F., Roux C. (2004). Contribution of RPB2 to multilocus phylogenetic studies of the Pezizomycotina (Euasco-mycetes, Fungi) with special emphasis on the lichen-forming Acarosporaceae and evolution of polyspory. Mol. Phylogenet. Evol..

[23] Rehner S. (2001). Primers for Elongation Factor 1-a (EF1-a). http://ocid.nacse.org/research/deephyphae/EF1primer.pdf.

[24] Rehner S.A., Buckley E. (2005). A Beauveria phylogeny inferred from nuclear ITS and EF1-α sequences: Evidence for cryptic diversification and links to *Cordyceps* teleomorphs. Mycologia.

[25] Glass N.L., Donaldson G.C. (1995). Development of primer sets designed for use with the PCR to amplify conserved genes from filamentous ascomycetes. Appl. Environ. Microbiol..

[26] Schmitt I., Crespo A., Divakar P.K., Fankhauser J.D., Herman-Sackett E., Kalb K., Nelsen M.P., Nelson N.A., Rivas-Plata E., Shimp A.D. (2009). New primers for promising single-copy genes in fungal phylogenetics and systematics. Persoonia.

[27] Nilsson R.H., Anslan S., Bahram M., Wurzbacher C., Baldrian P., Tedersoo L. (2019). Mycobiome diversity: High-throughput sequencing and identification of fungi. Nat. Rev. Microbiol..

[28] Hibbett D., Abarenkov K., Kõljalg U., Öpik M., Chai B., Cole J., Wang Q., Crous P., Robert V., Helgason T. (2016). Sequence-based classification and identification of fungi. Mycologia.

[29] Bulgarelli D., Rott M., Schlaeppi K., Van Themaat E.V.L., Ahmadinejad N., Assenza F., Rauf P., Huettel B., Reinhardt R., Schmelzer E. (2012). Revealing structure and assembly cues for *Arabidopsis* root-inhabiting bacterial microbiota. Nature.

[30] Lundberg D.S., Lebeis S.L., Paredes S.H., Yourstone S., Gehring J., Malfatti S., Tremblay T., Engelbrektson A., Kunin V., del Rio T.G. (2012). Defining the core *Arabidopsis thaliana* root microbiome. Nature.

[31] Knief C., Delmotte N., Chaffron S., Stark M., Innerebner G., Wassmann R., von Mering C., Vorholt J.A. (2012). Metaproteogenomic analysis of microbial communities in the phyllosphere and rhizosphere of rice. ISME J..

[32] Rastogi G., Sbodio A., Tech J.J., Suslow T.V., Coaker G.L., Leveau J.H.J. (2012). Leaf microbiota in an agroecosystem: Spatiotemporal variation in bacterial community composition on field-grown lettuce. ISME J..

[33] Bokulich N.A., Thorngate J.H., Richardson P.M., Mills D.A. (2014). Microbial biogeography of wine grapes is conditioned by cultivar, vintage, and climate. Proc. Natl. Acad. Sci. USA.

[34] Maignien L., Deforce E.A., Chafee M.E., Eren A.M., Simmons S.L. (2014). Ecological succession and stochastic variation in the assembly of *Arabidopsis thaliana* phyllosphere communities. mBio.

[35] Williams T.R., Moyne A.L., Harris L.J., Marco M.L. (2013). Season, irrigation, leaf age, and *Escherichia coli* inoculation influence the bacterial diversity in the lettuce phyllosphere. PLoS ONE.

[36] Dumbrell A.J., Ashton P.D., Aziz N., Feng G., Nelson M., Dytham C., Fitter A.H., Helgason T. (2011). Distinct seasonal assemblages of arbuscular mycorrhizal fungi revealed by massively parallel pyrosequencing. New Phytol..

[37] Gottel N.R., Castro H.F., Kerley M., Yang Z.M., Pelletier D.A., Podar M., Karpinets T., Uberbacher E., Tuskan G.A., Vilgalys R. (2011). Distinct microbial communities within the endosphere and rhizosphere of *Populus deltoides* roots across contrasting soil types. Appl. Environ. Microbiol..

[38] Peiffer J.A., Spor A., Koren O., Jin Z., Tringe S.G., Dangl J.L., Buckler E.S., Ley R.E. (2013). Diversity and heritability of the maize rhizosphere microbiome under field conditions. Proc. Natl. Acad. Sci. USA.

[39] Scholtysik A., Unterseher M., Otto P., Wirth C. (2012). Spatio-temporal dynamics of endophyte diversity in the canopy of European ash (*Fraxinus excelsior*). Mycol. Prog..

[40] Schlegel M., Queloz V., Sieber T.N. (2018). The endophytic mycobiome of European ash and sycamore maple leaves–geographic patterns, host specificity and influence of ash dieback. Front. Microbiol..

[41] Bialek R., González G.M., Begerow D., Zelck U.E. (2005). Coccidioido mycosis and blastomycosis: Advances in molecular diagnosis. FEMS Immunol. Med. Microbiol..

[42] Davydenko K., Vasaitis R., Stenlid J., Menkis A. (2013). Fungi in foliage shoots of *Fraxinus excelsior* in eastern Ukraine: A first report on Hymenoschyphus pseudoalbidus. For. Pathol..

[43] Bakys R., Vasaitis R., Barklund P., Thomsen I.M., Stenlid J. (2009). Occurrence and pathogenicity of fungi in necrotic and nonsymptomatic shoots of declining common ash (*Fraxinus excelsior*) in Sweden. Eur. J. For. Res..

[44] Kowalski T., Kraj W., Bednarz B. (2016). Fungi on stems and twigs in initial and advanced stages of dieback of European ash (*Fraxinus excelsior*) in Poland. Eur. J. For. Res..

[45] Cross H., Sønstebø J.H., Nagy N.E., Timmermann V., Solheim H., Børja I., Kauserud H., Carlsen T., Rzepka B., Wasak K. (2017). Fungal diversity and seasonal succession in ash leaves infected by the invasive ascomycete *Hymenoscyphus fraxineus*. New Phytol..

[46] Agostinelli M. (2018). Fungal Assemblages in Forest Trees—Influence of External and Internal Conditions. Ph.D. Thesis.

[47] Cleary M., Nguyen D., Marčiulynienė D., Berlin A., Vasaitis R., Stenlid J. (2016). Friend or foe? Biological and ecological traits of the European ash dieback pathogen *Hymenoscyphus fraxineus* in its native environment. Sci. Rep..

[48] Kosawang C., Amby D.B., Bussaban B., Mckinney L.V., Xu J., Kjær E.D., Collinge D.B., Nielsen L.R. (2018). Fungal communities associated with species of *Fraxinus* tolerant to ash dieback and their potential for biological control. Fungal Biol..

[49] Murphy B.R., Batke S.P., Doohan F.M., Hodkinson T.R. (2015). Media manipulations and the culture of beneficial fungal root endophytes. Int. J. Biol..

[50] Hodkinson T.R., Waldren S., Parnell J.A.N., Kelleher C.T., Salamin K., Salamin N. (2007). DNA banking for plant breeding, biotechnology and biodiversity evaluation. J. Plant Res..

[51] Perdereau A.C., Kelleher C.T., Douglas G.C., Hodkinson T.R. (2014). High levels of gene flow and genetic diversity in Irish populations of *Salix caprea* L. inferred from chloroplast and nuclear SSR markers. BMC Plant Biol..

[52] Hodkinson T.R., de Cesare M., Barth S. (2013). Nuclear SSR markers for Miscanthus, Saccharum, and related grasses (Saccharinae, Poaceae). Appl. Plant Sci..

[53] Lahiri A. (2020). Endophytic Diversity of *Fraxinus excelsior* L. (European Ash) and Its Interaction with the Dieback Pathogen *Hymenoscyphus fraxineus*. Ph.D. Thesis.

[54] Tamura K., Peterson D., Peterson N., Stecher G., Nei M., Kumar S. (2011). MEGA5: Molecular evolutionary genetic analysis using maximum likeli-hood, evolutionary distance, and maximum parsimony methods. Mol. Biol. Evol..

[55] Lozupone C., Lladser M.E., Knights D., Stombaugh J., Knight R. (2011). UniFrac: An effective distance metric for microbial community comparison. Microb. Ecol..

[56] Murphy B.R., Nieto L.M., Doohan F.M., Hodkinson T.R. (2015). Profundae diversitas: The uncharted genetic diversity in a newly studied group of fungal root endophytes. Mycology.

[57] Bakys R., Vasaitis R., Barklund P., Ihrmark K., Stenlid J. (2009). Investigations concerning the role of *Chalara fraxinea* in declining *Fraxinus excelsior*. Plant Pathol..

[58] Kowalski T., Łukomska A. (2005). Studies on *Fraxinus excelsior* L. dieback in Włoszczowa Forest Unit stands. Acta Agrobot..

[59] Parnell J., Curtis T. (2012). Webb’s an Irish Flora.

[60] Trapiello E., Schoebel C.N., Rigling D. (2017). Fungal community in symptomatic ash leaves in Spain. Balt. For..

[61] González V., Tello M.L. (2011). The endophytic mycota associated with *Vitis vinifera* in central Spain. Fungal Divers..

[62] Quilluam R.S., Jones D.L. (2012). Evidence for host-specificity of culturable fungal root endophytes from the carnivorous plant *Pinguicula vulgaris* (Common Butterwort). Mycol. Prog..

[63] Paul N.C., Lee H.B. (2014). First record of endophytic *Paraconiothyrium brasiliense* isolated from Chinese maple leaves in Korea. Korea J. Mycol..

[64] Kaur A., Raja H.A., Swenson D.C., Agarwal R., Deep G., Falkinham J.O., Oberlies N.H. (2016). Talarolutins A–D: Meroterpenoids from an endophytic fungal Isolate of *Talaromyces minioluteus*. Phytochemistry.

[65] Soca-Chafre G., Rivera-Orduña F.N., Hidalgo-Lara M.E., Hernandez-Rodriguez C., Marsch R., Flores-Cotera L.B. (2011). Molecular phylogeny and paclitaxel screening of fungal endophytes from Taxus globosa. Fungal Biol..

[66] Hayatgheibi H. (2013). Studies on the Microflora Associated with the SEEDS of European Ash (*Fraxinus excelsior*) and the Infection Biology of the Pathogen *Hymenoscyphus pseudoalbidus* Causing Ash Dieback. Master’s Thesis.

[67] Cleary M.R., Arhipova N., Gaitnieks T., Stenlid J., Vasaitis R. (2013). Natural infection of *Fraxinus excelsior* seeds by *Chalara fraxinea*. For. Pathol..

[68] Aveskamp M.M., de Gruyter J., Woudenberg J.H., Verkley G.J., Crous P.W. (2010). Highlights of the Didymellaceae: A polyphasic approach to characterise *Phoma* and related pleosporalean genera. Stud. Mycol..

