# Supplementary material for: Assessing Genotypic and Environmental Effects on Endophyte Communities of Fraxinus (Ash) Using Culture Dependent and Independent DNA Sequencing
Source: J Fungi (Basel). 2021 Jul 15;7(7):565. doi: 10.3390/jof7070565 (PMC8306109; doi:10.3390/jof7070565)
Supplement: Supplementary file 1 [file jof-07-00565-s001.zip › Supplement Figs R1 .pdf]

Journal of *Fungi*

MDPI

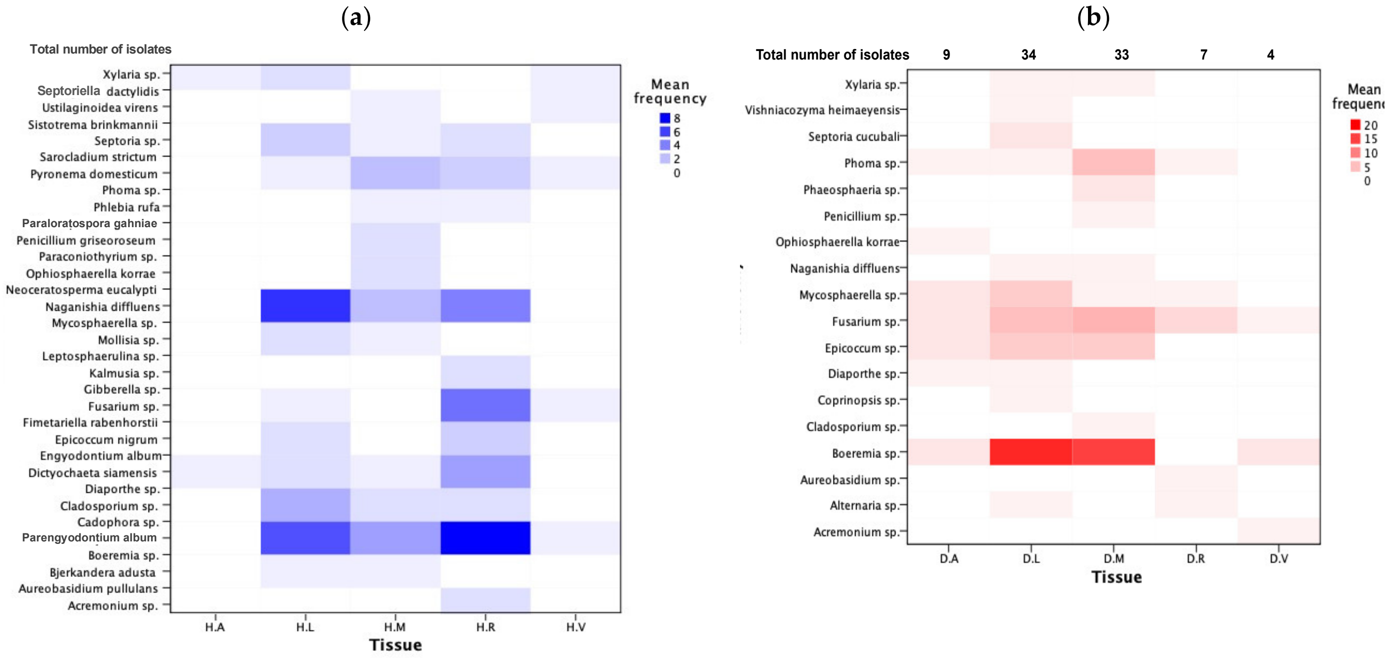


**Figure S1.** Heat-map for mean frequency of OTUs in different leaf tissue types. (**a**) In healthy ash leaf tissues sampled from Roosky. H.L = 4 cm above from the leaf base on the sides of lamina, H.M = middle lamina with midrib portion, H.R = healthy rachis, H.A = healthy apex, H.V = healthy veins (mostly the same as H.L only in 10 cm below apex); (**b**) In diseased ash leaf tissue sampled from Roosky. D.L = diseased leaf tissue position not fixed, D.R = diseased rachis, D.M = diseased midrib with laminar tissue, D.A = diseased apex, D.V = diseased veins.

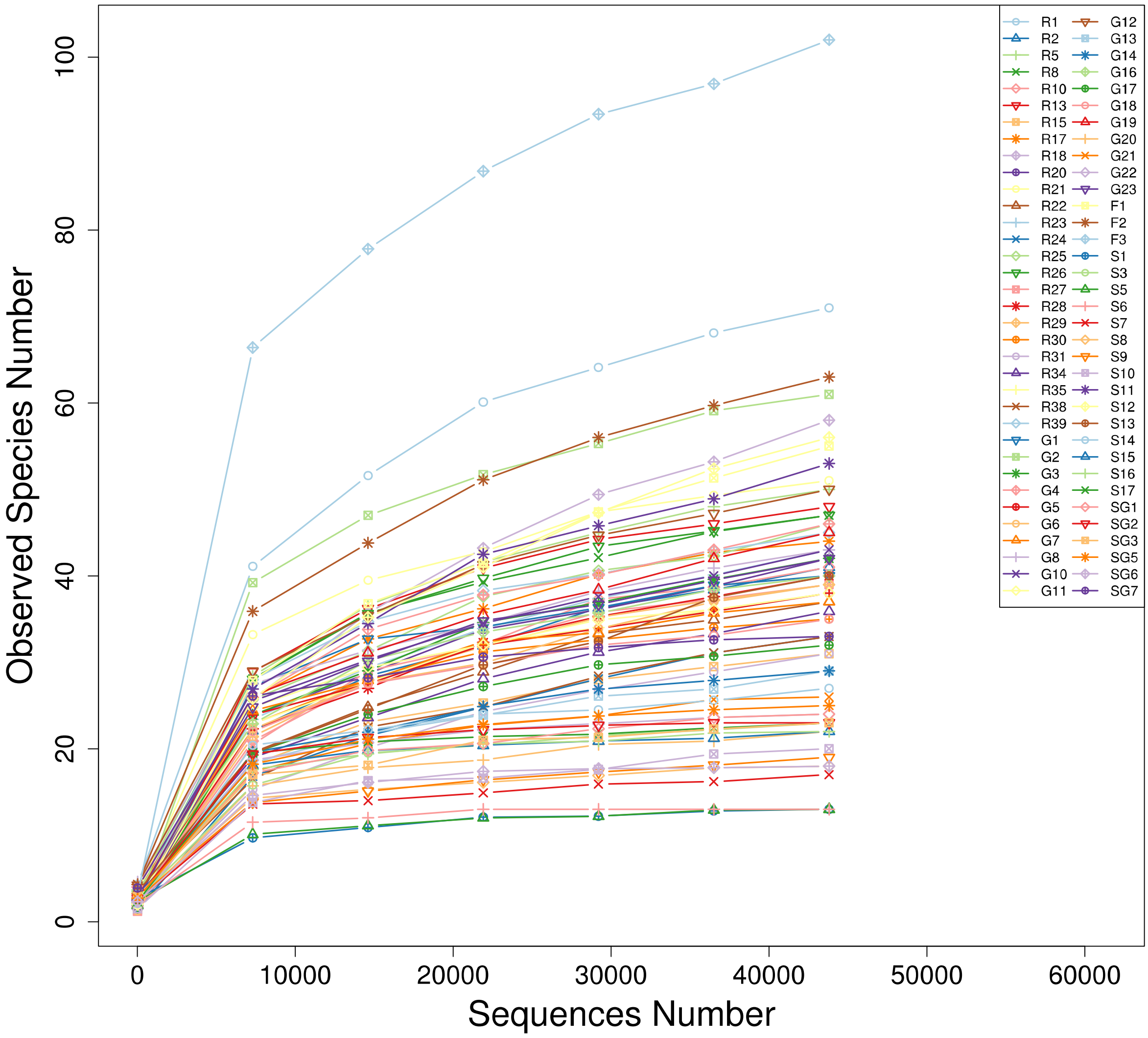


**Figure S2.** Rarefaction curves for all HTS samples. 70 samples each represented here by a separate line were included and most leveled off – thus most species (OTUs) are captured with this number of reads in each sample. Rarefaction categories are for 10; 7,309; 14,608; 21,907; 29,206; 36,505 and 43,804 sequence reads (x axis). R= samples from Roosky, G= samples from Glasnevin Botanic Garden, S= seed samples, F= France.

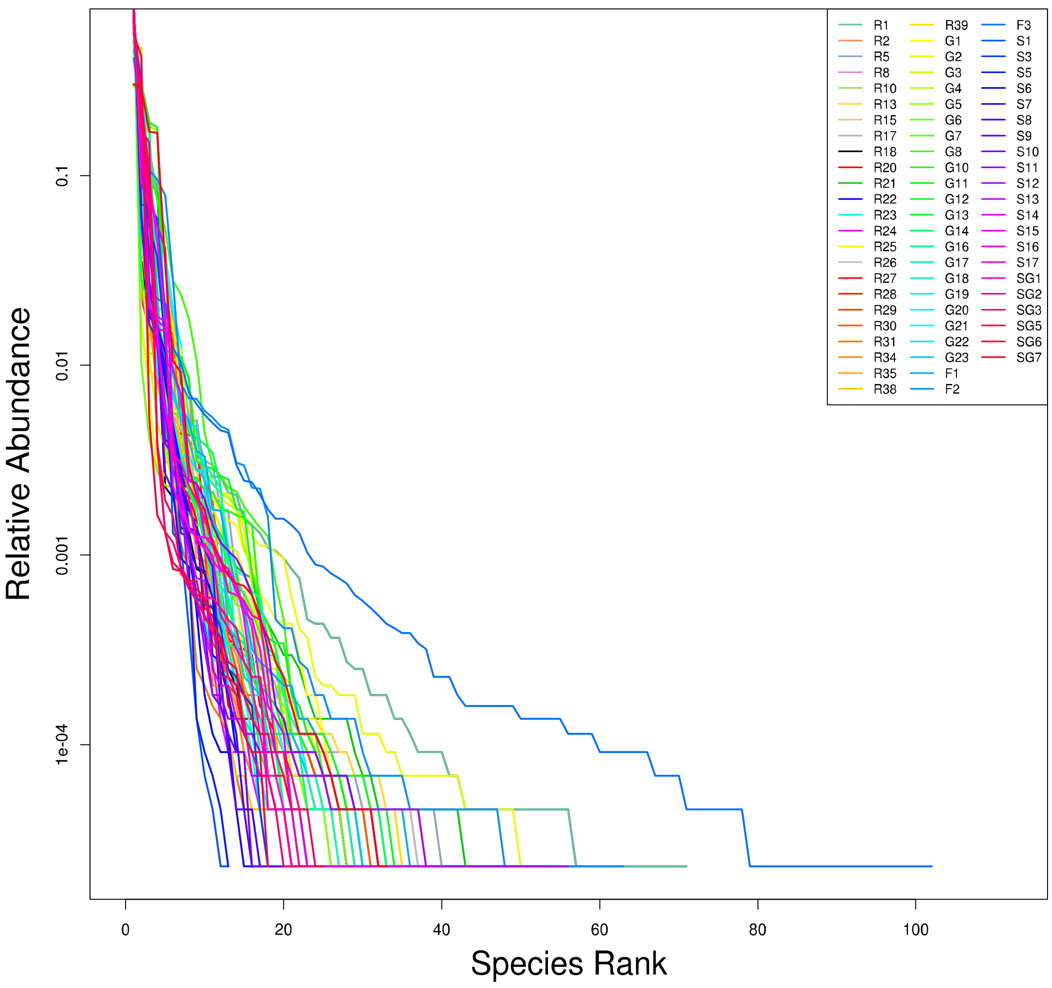


**Figure S3.** Rank abundance curves for all samples. Each sample is represented by a single line. R= samples from Roosky, G= samples from Glasnevin Botanic Garden, S= seed samples, F= France.

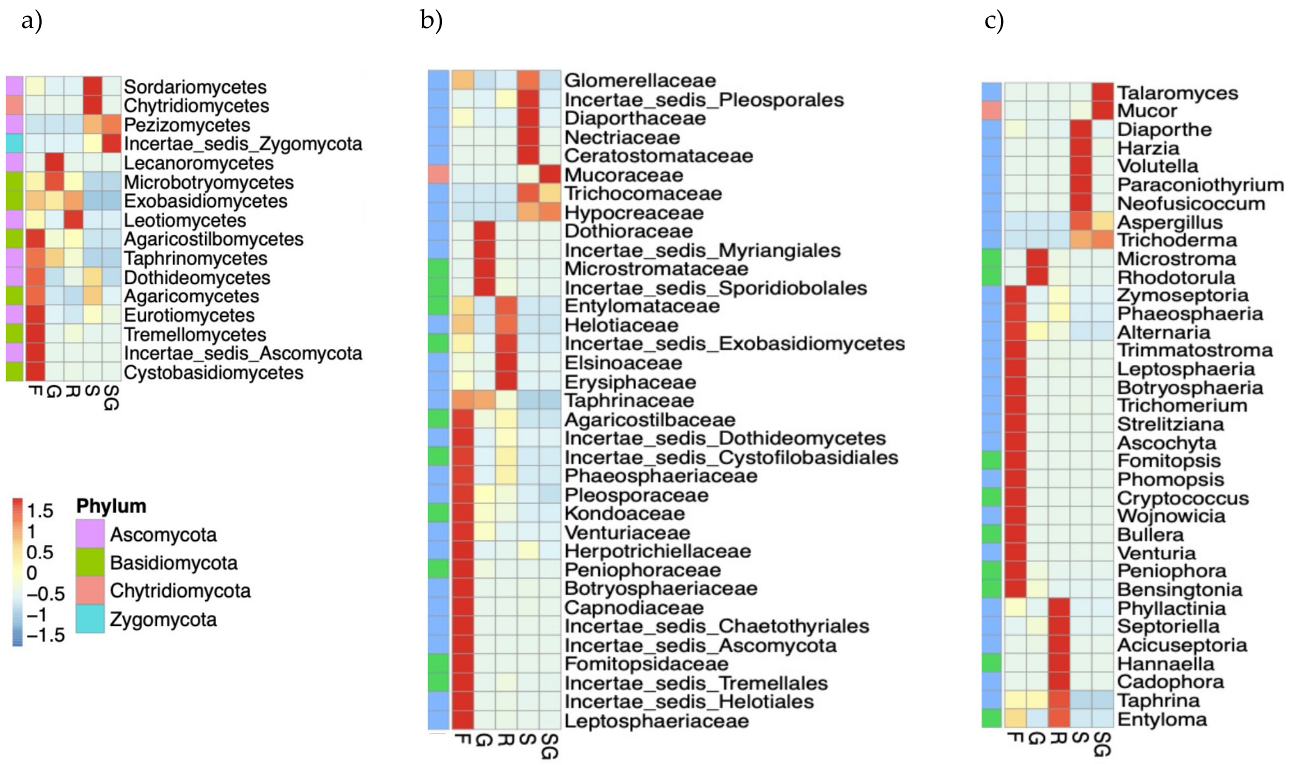


**Figure S4.** OTU abundance heat map categorized into phyla, classes, families or genera of the 70 samples screened with HTS of nrITS. a) classes; b) families; c) genera. Only common families and genera shown. Red shows high proportion of that group in the sample group and blue a low proportion (F=France, G=Glasnevin, R=Roosky, S=Seed Roosky, SG=Seed Glasnevin). The absolute value of 'z' represents the distance between the raw score and the mean of the standard deviation. 'Z' is negative when the raw score is below the mean, and vice versa.

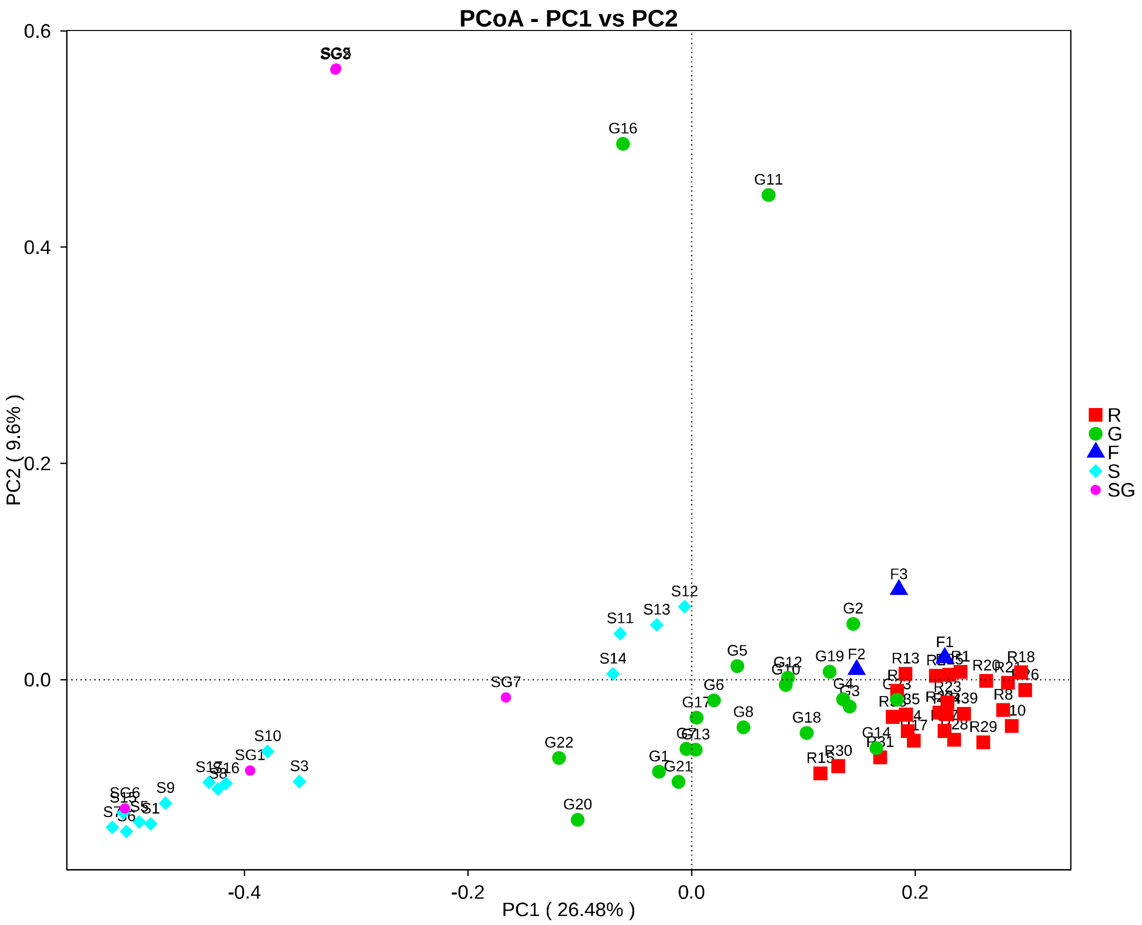


**Figure S5.** PCoA with unweighted unifrac distances. R= Roosky samples, G= Glasnevin samples, F= France sample, S= seed from Roosky samples, SG= seeds from Glasnevin samples. Percentage of variance explained by each axis shown.

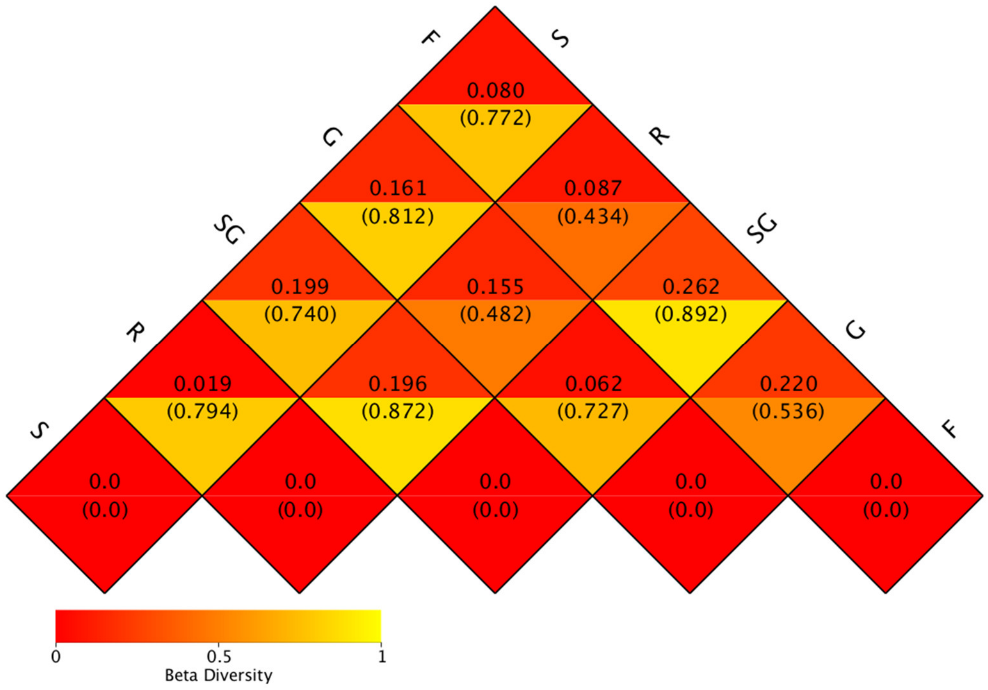


**Figure S6.** Heat-map for beta diversity. Each cell in the grid represents a pairwise dissimilarity coefficient between groups. Unweighted unfrac distances below the line; weighted unfrac distances above the line. All comparisons of beta diversity with weighted unifrac distances are significantly different with Tukey HSD at $p<0.05$ except G-F, SG-G, and S-R (in Wilcoxon tests all are significantly different except G-F, F-SG, SG-G and S-R). With unweighted unifrac distances only R-G, S-G, S-R, and SG-R are significantly different with Tukey HSD (in Wilcoxon tests R-G, S-G, S-R, and SG-R, F-R and SG-G are significantly different at $p<0.05$ level).
