# Supplementary material for: Assessing Genotypic and Environmental Effects on Endophyte Communities of Fraxinus (Ash) Using Culture Dependent and Independent DNA Sequencing
Source: J Fungi (Basel). 2021 Jul 15;7(7):565. doi: 10.3390/jof7070565 (PMC8306109; doi:10.3390/jof7070565)
Supplement: Supplementary file 1 [file jof-07-00565-s001.zip › Supplementary tablesR1Revised.pdf]

Journal of Fungi

MDPI

Table S1. Ash leaflets, seed and roots collected for fungal isolations, DNA extraction and HTS. Leaves and seed collected from Roosky, Co. Roscommon; leaves only collected from Glasnevin Botanic Garden, Co. Dublin and Lake Annecy France. Roots collected from Kinsealy, Co. Dublin genebank only.

No.	Code or name	Additional info
**Roosky leaves**	***Fraxinus excelsior***	**Plot number**
1	A	13
2	A1.a	5
3	A1.b	5
4	A1.c	5
5	B	20
6	C	7
7	D1.a	12
8	D1.b	12
9	D1.c	12
10	E	9
11	E1.a	11
12	E1.b	11
13	F1.a	21
14	F1.b	21
15	G.a	24
16	G.b	24
17	G1.a	30
18	G1.b	30
19	H	25
20	H1.a	1
21	H1.b	1
22	J	29
23	J1	18
24	K	2
25	K1.b	10
26	L	14
27	L1	33
28	M	31
29	M1	15
30	O1	27
31	Q1	37
32	R1.a	28
33	R1.b	28
34	S1.a	19
35	S1.b	19
36	T1.a	32
37	T1.b	32
38	T1.c	32
39	U1	36
40	Y	23

**Roosky seed**	***Fraxinus excelsior***	**Plot number**
1	A	7
2	B	13
3	B	15

4	B	17
5	B (Irish)	16
6	C	12
7	D	4
8	D1	9
9	E1	3
10	G	2
11	G1.b	14
12	H	10
13	K	8
14	Q	11
15	U	5
16	U1	1
17	Z	6

**Glasnevin leaf**	**Taxon**	**Accession**
1	*Fraxinus texensis*	011046
2	*Fraxinus xanthoxyloides*	2003.0735
3	*Fraxinus glabra*	1909.011054
4	*Fraxinus mandshurica*	1934.011053
5	*Fraxinus potamophila*	1932.011031
6	*Fraxinus americana*	2001.1739
7	*Fraxinus pubinervis*	1954.011044
8	*Fraxinus angustifolia*	011028
9	*Fraxinus excelsior*	2000.3637A
10	*Fraxinus ornus*	1977.0039
11	*Fraxinus ornus*	2005.0652
12	*Fraxinus excelsior (‘Di-*	011040
13	*Fraxinus dipetala*	011038
14	*Fraxinus excelsior* (or-	011029
15	*Fraxinus* sp..	2013.0214
16	*Fraxinus ornus*	1969.011033
17	*Fraxinus ornus*	011034
18	*Fraxinus numidica*	1904.011032
19	*Fraxinus angustofolia*	011074
20	*Fraxinus* sp.	2006.1641
21	*Fraxinus* sp. (greybud)	*s.n.*
22	*Fraxinus excelsior* ‘Pen-	011048
23	*Fraxinus pennsylvanica*	1889.011030

**France leaf**	***Fraxinus excelsior***	**Location**
1	S1	Lake Annecy, France
2	S2	Lake Annecy, France
3	S3	Lake Annecy, France

**Roots**	***Fraxinus excelsior***	**Accession**
1	RI	RI
2	R2	R2
3	R3	R3

Table S2. Samples selected for HTS Ilumina analysis and their quantity of total DNA (ngul-1)

Place	Plot	Country	Provenance	Tissue	Name	Name/plot	DNA yield
R	13	IE	ENN	Leaf	R1 *	A	93.1
R	5	BE	HOG	Leaf	R2	A1.A	90.8
R	20	IE	DON	Leaf	R5	B	81.9
R	12	IE	Control	Leaf	R8	D1.C	79.7
R	11	DE	FAR	Leaf	R10	E1.B	83.4
R	21	DE	KAR	Leaf	R13	F.O	39.2
R	24	GB	LOC	Leaf	R15	G.B	67
R	30	DK	RAV	Leaf	R17	G1.B	98.9
R	25	FR	VSP	Leaf	R18	H	63.5
R	1	DK	BRE	Leaf	R20	H1.B	91.3
R	29	FR	SAM	Leaf	R21	I	26
R	18	IT	ABE	Leaf	R22	I1	111.7
R	2	FR	ATH	Leaf	R23	K	50.6
R	10	IT	VAP	Leaf	R24	K1	73.3
R	14	FR	LAR	Leaf	R25	L	72.4
R	33	IT	CAD	Leaf	R26	L1	57.7
R	31	GB	WYW	Leaf	R27	M	69.2
R	15	IT	MON	Leaf	R28	M1	51.1
R	27	PO	WLO	Leaf	R29	O1	137
R	37	PO	WLO	Leaf	R30	O1	66
R	28	LT	KAL	Leaf	R31	R1.A	130.1
R	19	LT	ZEI	Leaf	R34	S1.B	76.4
R	32	CZ	RAB	Leaf	R35	T1.A	139.9
R	36	PO	MIR	Leaf	R38	U1	85.4
R	23	FR	SAG	Leaf	R39	Y	55.3
G	-	a	-	Leaf	G1 **	*F. texensis*	75.9
G	-	b	-	Leaf	G2	*F. xanthoxy-*	52.4
G	-	c	-	Leaf	G3	*F. glabra*	78.9
G	-	d	-	Leaf	G4	*F. mandshurica*	138.2
G	-	e	-	Leaf	G5	*F. potamophila*	27.7
G	-	f	-	Leaf	G6	*F. americana*	144.2
G	-	g	-	Leaf	G7	*F. pubinervis*	117.1
G	-	h	-	Leaf	G8	*F. angustifolia*	84.5
G	-	i	-	Leaf	G10	*F. ornus*	145.6
G	-	j	-	Leaf	G11	*F. ornus*	71.5
G	-	k	-	Leaf	G12	*F. excelsior*	53.6
G	-	l	-	Leaf	G13	*F. dipetala*	117
G	-	m	-	Leaf	G14	*F. excelsior*	125.4
G	-	n	-	Leaf	G16	*F. ornus*	102.5
G	-	o	-	Leaf	G17	*F. ornus*	72.5
G	-	p	-	Leaf	G18	*F. numidica*	42.1
G	-	q	-	Leaf	G19	*F. angustifolia*	63.5
G	-	r	-	Leaf	G20	*Fraxinus sp.*	56.4
G	-	s	-	Leaf	G21	*Fraxinus* (un-	140.1
G	-	t	-	Leaf	G22	*F. excelsior*	21.5
G	-	u	-	Leaf	G23	*F. pennsylvan-*	78.6
F	-	-	ABL	Leaf	F1 °	*Fraxinus* sp.	24.2
F	-	-	ABL	Leaf	F2	*Fraxinus* sp.	27.1
F	-	-	ABL	Leaf	F3	*Fraxinus* sp.	41.2
R	3	FR	SPD	Seed	S1 w	O (2.3)	93
R	20	IE	DON	Seed	S3	B (6.6)	102.6
R	13	IE	ENN	Seed	S5	A (5.4)	31.1
R	25	FR	VSP	Seed	S6	H (5.5)	58.2
R	20	IE	DON	Seed	S7	B (1.3)	39.2
R	2	FR	ATH	Seed	S8	K (1.6)	54.4
R	12	NL	VCS	Seed	S9	D1 (4.4)	98.8
R	9	GB	SET	Seed	S10	E (2.5)	39.4
R	4	FR	DOU	Seed	S11	Z (2.2)	48.4

R	17	IE	Control	Seed	S12	D (3.5)	247.3
R	7	IE	CUR	Seed	S13	C (2.2)	34.5
R	6	FR	SPF	Seed	S14	U (5.5)	47.9
R	36	PO	MIR	Seed	S15	U1 (2.4)	39.7
R	30	DK	RAV	Seed	S16	G1 (b)	51.8
R	24	GB	LOC	Seed	S17	G (5.4)	32.8
G	-	v	-	Seed	SG1 ◎	v	121
G	-	n	-	Seed	SG2	*F.ornus*	98.3
G	-	w	-	Seed	SG3	*F.ornus*	22.5
G	-	s	-	Seed	SG5	x	66.2
G	-	e	-	Seed	SG6	*F. potamophila*	34.2
G	-	d	-	Seed	SG7	*F. mandshurica*	11.9

*R1-R39 = Leaflets from Roosky; **G1-G23= Leaflets from Glasnevin; °F1-F3= Leaflets from France; ◎S1-S17= Seed extract from Roosky; ◎SG1-SG7=Seed extracts from Glasnevin; Belgium – BE, Czech republic – CZ, Denmark – DK, France – FR, Germany – DE, Ireland – IE, Italy – IT, Lithuania – LT, Netherland – NL, Poland – PO, UK – GB; Abeton (IT) - ABE, Athis (FR) - ATH, Au BoutduLac – ABL, Bregentved (DK)-BRE, Cadore (IT)-CAD, Currachase (IE)- CUR, Donadea (IR)-DON, Dourdan – DOU, Enniskillen (IE)-ENN, Farchau (DE)-FAR, Hoge Bos (BE)-HOG, Kalsiadorys (LT)- KAL, Karlsruhe (DE) -KAR, La Romagne (FR)–LAR, Loch Tay (GB)-LOC, Mircze (PO)- MIR, Monte Lessini (IT)– MON, Rabstejn (CZ)- RAB, Ravenholt (DK)– RAV, Saint Gatien (FR)- SAG, Saint Martin (FR) – SAM, Settrington (GB)- SET, St Paul De Salers – SPD, St. Pierre des fleurs – SPF,Vaartbos Com.seed (NL) – VCS, Val Saint Pierre (FR) – VSP, Valle Pesio (IT)– VAP, Wloszczowa (PO) – WLO, Wytham Wood (GB)– WYW, Zeimelis (LT)- ZEI; accession number XX.011046 – a, 2003.0735 – b, 1909.011054 – c, 1934.011053 – d, 1932.011031 – e, 2001.1739 – f, 1954.011044 – g, XX.011028 – h, 1977.0039 – i, 2005.0652 – j, XX.011040 – k, XX.011038 – l, XX.011029 – m, 1969.011033 – n, XX.011034 – o, 1904.011032 – p, XX.011074 – q, 2006.1641 – r, GPS -78 – s, XX.011048 – t, 1889.011030 – u. GPS-91 – v, GPS-83 – w, GPS 81- x.

Table S3. Primer information for each DNA barcoding locus used (nrITS, *tef* and LSU).

Primer	Primer sequence	Reference
**Forward**		
ITS-1	5′ TCCGTAGGTGAACCTGCGG 3′	White *et al.* (1990)
ITS-1F	5′ CTTGGTCATTTAGAGGAAGTAA 3′	Gardes and Bruns (1993)
ITS-5	5′ GGAAGTAAAAGTCGTAACAAGG 3′	White *et al.* (1990)
LROR (LSU)	5′ ACCCGCTGAACTTAAGC 3′	Vilgalys and Hester (1990)
Tef-983	5′ GCYCCYGGHCAYCGTGAYTTYAT 3′	Rehner and Buckley (2005)
**Reverse**		
ITS-4	5′ TCCTCCGCTTATTGATATGC 3′	White *et al.* (1990)
LR5 (LSU)	5′ TCCTGAGGGAAACTTCG 3′	Vilgalys and Hester (1990)
Tef-1576	5′ ACHGTRCCRATACCACCRATCTT 3′	Rehner and Buckley (2005)

Table S4. Frequencies for each of the OTUs at the study sites. Roosky and Glasnevin are leaf endophytes; Kinsealy are root endophytes.

No.	OTU	GenBank Accession	Tissue specific-	Roosky, L	Glasnevin, L	Kinsealy, R
				Frequencies of OTUs recovered		
1	*Acremonium alternatum*	+	R	-	-	1*
2	*Acremonium* sp.	MZ509281	L	2	2	-
3	***Akanthomyces attenuatus***	+	L	-	1	-
4	*Akanthomyces lecanii*	MZ509282; MZ509283	R	-	-	2
5	***Akanthomyces muscarium***	+	L	-	1	-
6	*Alternaria humuli*	+	L	1	-	-
7	*Alternaria* sp.	MZ509284	L	1	-	-
8	***Aspergillus versicolor***	MZ509285	L	-	1	-
9	*Aureobasidium pullulans*	MZ509286; MZ509287	L	1	1	-
10	*Aureobasidium* sp.	+	L	1	1	-
11	*Bjerkandera adusta*	MZ509288	L	1	-	-
12	*Boeremia exigua*	MZ509289; MZ509290	L	58	2	-
13	*Boeremia hedericola*	MZ509291	L	5	-	-
14	*Boeremia* sp.	+	L	1	-	-
15	*Boeremia strasseri*	MZ509292	L	2	-	-
16	*Boeremia trachelospermi*	+	L	6	-	-
17	*Botrytis cinerea*	MZ509293	L	1	-	-
18	*Cadophora meredithiae*	+	R	-	-	1
19	*Cadophora* sp.	MZ509294	Both R	1	-	5
20	*Cladosporium allicinum*	MZ509295	L	2	-	-
21	*Cladosporium cladosporioides*	MZ509296	Both R	1	-	1
22	***Cladosporium floccosum***	+	L	-	1	-
23	*Cladosporium herbarum*	MZ509297; MZ509298	L	1	-	-
24	***Cladosporium kenpeggii***	MZ509299	L	-	1	-
25	***Cladosporium perangustum***	+	L	-	1	-
26	*Cladosporium* sp.	MZ509300; MZ509301	L	6	6	-
27	*Cladosporium westerdijkiae*	+	R	-	-	1
28	***Collembolispora aristata***	MZ506894	L	-	1	-
29	***Colletotrichum godetiae***	MZ509302	L	-	1	-
30	*Coprinopsis* sp.	MZ509303	L	1	-	-
31	***Cystobasidium slooffiae***	MZ509304	L	-	1	-
32	*Dactylonectria alcacerensis*	MZ509305	R	-	-	1
33	*Dactylonectria hordeicola*	MZ509306	R	-	-	6
34	*Dactylonectria macrodidyma*	MZ509307	R	-	-	20
35	*Dactylonectria torresensis*	MZ509308	R	-	-	2
36	***Diaporthe cotoneastri***	+	L	-	2	-
37	*Diaporthe passiflorae*	MZ509309	L	5	-	-
38	*Diaporthe rudis*	MZ509310	L	1	2	-
39	*Diaporthe* sp.	MZ509311	L	2	-	-
40	*Diaporthe viticola*	MZ509312	L	2	2	-
41	*Dictyochaeta siamensis*	MZ509313; MZ506890	L	1	-	-
42	*Ectophoma multirostrata*	MZ509314	L	2	-	-
43	*Epichloe typhina*	MZ509315	R	-	-	1
44	*Epicoccum nigrum*	MZ509316	Both R	6	-	2
45	*Epicoccum* sp.	MZ509317	L	4	-	-

46	***Eutypa spinosa***	MZ509318	L	-	1	-
47	***Exophiala oligosperma***	MZ509319	L	-	2	-
48	*Fimetariella rabenhorstii*	MZ509320	L	1	-	-
49	*Fusarium avenaceum*	MZ509321; MZ509322	L	1	1	-
50	*Fusarium culmorum*	MZ509323	Both R	-	1	7
51	*Fusarium lateritium*	MZ509324	L	12	8	-
52	*Fusarium oxysporum*	MZ509325	Both R	1	2	5
53	*Fusarium proliferatum*	MZ509326	L	1	2	-
54	*Fusarium* sp.	MZ509327	L	2	1	-
55	*Fusarium tricinctum*	MZ509328	L	1	-	-
56	*Gibberella* sp.	+	L	1	-	-
57	***Gloniopsis calami***	+	L	-	1	-
58	*Harzia velata*	+	R	-	-	2
59	*Hydropisphaera* sp.	MZ509329	R	-	-	1
60	*Ilyonectria destructans*	MZ509330	R	-	-	1
61	*Ilyonectria radicicola*	MZ509331	R	-	-	6
62	*Ilyonectria robusta*	MZ509332	R	-	-	2
63	*Ilyonectria* sp.	MZ509333	R	-	-	5
64	*Juxtiphoma eupyrena*	MZ509334	R	-	-	2
65	*Kalmusia* sp.	+	L	1	-	-
66	*Lecanicillium* sp.	+	R	-	-	1
67	*Leptosphaerulina australis*	+	L	1	-	-
68	*Leptosphaerulina trifolii*	MZ509337	L	1	-	-
69	***Laetisaria roseipellis***	MZ509335	L	-	1	-
70	***Laetisaria* sp.**	MZ509336	L	-	2	-
71	***Meyerozyma guilliermondii***	MZ509338	L	-	2	-
72	*Mollisia* sp.	+	L	1	-	-
73	*Mycochaetophora sp.*	+	R	-	-	1
74	*Mycosphaerella coacervata*	MZ509339	L	28	3	-
75	*Mycosphaerella sp.*	MZ509340	L	3	-	-
76	*Naganishia diffluens*	+	L	4	-	-
77	*Nectria* sp.	MZ509341	R	-	-	1
78	*Neoceratosperma eucalypti*	+	L	1	-	-
79	***Neofabraea vagabunda***	MZ509342	L	-	1	-
80	*Neonectria candida*	MZ509343	R	-	-	1
81	*Neonectria punicea*	+	R	-	-	2
82	*Neonectria radicicola*	MZ509344	R	-	-	1
83	*Neonectria* sp.	MZ509345; MZ509346	R	-	-	9
84	*Ophiocordyceps crassispora*	+	R	-	-	2
85	*Ophiosphaerella korrae*	MZ506893	L	2	-	-
86	*Paraconiothyrium* sp.	MZ509347	L	1	-	-
87	*Paraloratospora gahniae*	MZ509348	L	2	-	-
88	*Parengyodontium album*	MZ509349	L	4	2	-
89	***Penicillium expansum***	+	L	-	1	-
90	*Penicillium griseoroseum*	MZ509350	L	1	-	-
91	*Penicillium* sp.	MZ509351	L	1	-	-
92	*Penicillium spathulatum*	MZ509352	R	-	-	1
93	*Phaeosphaeria pontiformis*	MZ509354	L	1	-	-
94	*Phlebia rufa*	MZ509355	L	1	-	-

95	*Phoma* sp.	MZ509353; MZ509356	L	12	2	-
96	***Phomopsis* sp.**	MZ509357	L	-	2	-
97	*Phomopsis velata*	MZ509358	L	1	1	-
98	*Psiloglonium* sp.	MZ509359; MZ509360	R	-	-	4
99	*Pyronema domesticum*	MZ509361; MZ506892	L	1	1	-
100	*Sarocladium strictum*	MZ509362; MZ509363	L	2	-	-
101	*Septoria convolvuli*	+	L	1	-	-
102	*Septoria cucubali*	MZ509364; MZ506891	L	5	-	-
103	***Septoria lepidiicola***	+	L	-	1	-
104	***Septoria protearum***	MZ509365; MZ509366	L	-	1	-
105	*Septoriella dactylidis*	MZ509368	L	1	-	-
106	*Sistotrema brinkmannii*	MZ509369	L	1	-	-
107	*Trichoderma viride*	MZ509370	R	-	-	3
108	*Ustilaginoidea virens*	MZ509371	L	1	-	-
109	***Ustilago filiformis***	+	L	-	2	-
110	*Vishniacozyma heimaeyensis*	MZ509372	L	1	-	-
111	***Vuilleminia coryli***	+	L	-	1	-
112	*Xylaria* sp.	MZ509373	L	5	-	-
**Total no. of isolates, total no. OTUs**				**219, 61**	**69, 41**	**100, 32**

Bold font = other *Fraxinus* species only (not *F. excelsior*); Tissue specificity: L=Leaf only, R=Root only

Genbank absent from some taxa (indicated with +) because they did not reach quality threshold for submission (available on request).

Table S5. Total number of isolates and OTUs within each class and family from all leaf and root endophytes. Asco.= Ascomycota; Basid.= Basidiomycota.

Class (Division, Subdivision)	Isolates	OTUs	Family	Isolates	OTUs
Dothideomycetes (Asco., Pezizomycotina)	195	40	Aspergillaceae	5	5
Eurotiomycetes (Asco., Pezizomycotina)	7	6	Bionectriaceae	1	1
Leotiomycetes (Asco., Pezizomycotina)	6	6	Bulleribasidiaceae	1	1
Pezizomycetes (Asco., Pezizomycotina)	2	1	Ceratostomataceae	2	1
Sordariomycetes (Asco., Pezizomycotina)	162	47	Chaetosphaeriaceae	1	1
Saccharomycetes (Asco., Saccharomycotina)	2	1	Cladosporiaceae	21	8
Agaricomycetes (Basid., Agaricomycotina)	8	7	Clavicipitaceae	1	1
Tremellomycetes (Basid., Agaricomycotina)	5	2	Cordycipitaceae	11	5
Cystobasidiomycetes (Basid., Pucciniomycotina)	1	1	Corticiaceae	4	3
Ustilaginomycetes (Basid., Ustilaginomycotina)	2	1	Cystobasidiaceae	1	1
-	-	-	Debaryomycetaceae	2	1
-	-	-	Dermateaceae	2	2
-	-	-	Diaporthaceae	20	7
-	-	-	Diatrypaceae	1	1
-	-	-	Didymellaceae	104	10
-	-	-	Didymosphaeriaceae	2	2
-	-	-	Dothioraceae	4	2
-	-	-	Filobasidiaceae	4	1
-	-	-	Glomerellaceae	1	1
-	-	-	Helotiales	9	4
-	-	-	Herpotrichiellaceae	2	1
-	-	-	Hydnaceae	1	1

-	-	-	Hypocreaceae	3	1
-	-	-	Hypocreales	20	7
-	-	-	Hysteriaceae	5	2
-	-	-	Lasiosphaeriaceae	1	1
-	-	-	Meruliaceae	2	2
-	-	-	Mycosphaerellaceae	45	7
-	-	-	Nectriaceae	89	17
-	-	-	Ophiocordycipitaceae	2	1
-	-	-	Phaeosphaeriaceae	6	4
-	-	-	Pleosporaceae	4	4
-	-	-	Psathyrellaceae	1	1
-	-	-	Pyronemataceae	2	1
-	-	-	Sarocladiaceae	2	1
-	-	-	Sclerotiniaceae	1	1
-	-	-	Ustilaginaceae	2	1
-	-	-	Xylariaceae	5	1

Table S6. OTU frequencies obtained from all sampled leaf tissues from all provenances in the Roosky provenance trial for *Fraxinus excelsior.*

	Country										
OTU	BE	CZ	DK	FR	DE	IE	IT	LT	NL	PO	GB
	Count										
Acremonium sp.	-	**1**	-	-	**1**	-	-	-	-	-	-
Alternaria humuli	-	-	-	-	-	-	-	-	-	**1**	-
Alternaria sp.	-	**1**	-	-	-	-	-	-	-	-	-
Aureobasidium pullulans	-	-	-	-	-	-	**1**	-	-	-	-
Aureobasidium sp.	-	-	**1**	-	-	-	-	-	-	-	-
Bjerkandera adusta	-	-	-	-	-	-	-	-	-	-	**1**
Boeremia exigua	1	**8**	**9**	**6**	**6**	**5**	**8**	**3**	**6**	**3**	**3**
Boeremia hedericola	-	**2**	-	-	**1**	-	**1**	-	-	**1**	-
Boeremia sp.	-	-	-	-	-	-	-	**1**	-	-	-
Boeremia strasseri	-	-	-	**1**	-	-	-	-	-	-	**1**
Boeremia trachelospermi	-	-	-	**3**	**1**	-	**1**	-	-	**1**	-
Botrytis cinerea	-	-	-	-	-	-	-	**1**	-	-	-
Cadophora sp.	-	-	-	-	-	**1**	-	-	-	-	-
Cladosporium allicinum	-	-	**1**	-	-	-	-	-	-	-	-
Cladosporium cladospori-	-	-	-	-	-	**1**	-	-	-	-	-
Cladosporium herbarum	-	-	-	-	-	-	-	-	-	-	**1**
Cladosporium sp.	-	-	**1**	-	-	**1**	-	**1**	**1**	-	**2**
Coprinopsis sp.	-	-	-	-	-	-	-	**1**	-	-	-
Diaporthe passiflorae	-	-	**1**	-	**1**	**1**	-	**1**	-	**1**	-
Diaporthe rudis	-	-	-	-	**1**	-	-	-	-	-	-
Diaporthe sp.	-	-	-	-	-	**1**	-	-	-	-	**1**
Diaporthe viticola	-	-	-	-	-	**1**	**1**	-	-	-	-
Dictyochaeta siamensis	-	-	-	-	-	-	-	-	**1**	-	-

Ectophoma multirostrata	-	-	**1**	-	**1**	-	-	-	-	-	-
Epicoccum nigrum	**1**	-	-	-	-	**1**	**1**	**1**	-	-	**2**
Epicoccum sp.	-	-	-	-	-	**1**	**1**	**1**	-	-	**1**
Fimetariella rabenhorstii	-	-	-	-	-	-	-	-	**1**	-	-
Fusarium avenaceum	-	-	-	-	-	**1**	-	-	-	-	-
Fusarium lateritium	-	-	**1**	**1**	**4**	**1**	**1**	-	**2**	**1**	**1**
Fusarium oxysporum	-	-	-	-	-	-	-	-	-	-	**1**
Fusarium proliferatum	-	-	-	-	-	-	-	-	-	**1**	-
Fusarium sp.	-	-	**1**	**1**	-	-	-	-	-	-	-
Fusarium tricinctum	-	-	-	-	-	-	**1**	-	-	-	-
Gibberella sp.	-	-	-	-	-	-	-	-	-	**1**	-
Kalmusia sp.	-	-	-	**1**	-	-	-	-	-	-	-
Leptosphaerulina australis	-	-	**1**	-	-	-	-	-	-	-	-
Leptosphaerulina trifolii	-	-	-	**1**	-	-	-	-	-	-	-
Mollisia sp.	-	-	-	-	-	-	-	-	-	**1**	-
Mycosphaerella coacervata	**2**	**2**	**2**	**7**	-	**3**	**5**	**3**	**1**	**1**	**3**
Mycosphaerella sp.	**1**	-	-	-	**1**	-	-	-	-	-	**1**
Naganishia diffluens	**2**	-	-	**1**	-	-	-	-	-	-	**1**
Neoceratosperma eucalypti	-	**1**	-	-	-	-	-	-	-	-	-
Ophiosphaerella korrae	**2**	-	-	-	-	-	-	-	-	-	-
Paraconiothyrium sp.	-	-	-	**1**	-	-	-	-	-	-	-
Paroloratospora gahniae	-	-	**1**	-	-	**1**	-	-	-	-	-
Parengyodontium album	-	-	-	**2**	-	**1**	-	**1**	-	-	-
Penicillium griseoroseum	-	-	-	-	-	-	-	-	-	**1**	-
Penicillium sp.	-	**1**	-	-	-	-	-	-	-	-	-
Phaeosphaeria pontiformis	-	-	-	-	**1**	-	-	-	-	-	-
Phlebia rufa	**1**	-	-	-	-	-	-	-	-	-	-
Phoma sp.	**2**	**2**	**3**	-	**1**	**2**	-	-	**1**	-	**1**
Phomopsis velata	-	-	-	-	-	-	-	-	**1**	-	-
Pyronema domesticum	-	-	-	-	-	**1**	-	-	-	-	-
Sarocladium strictum	-	-	-	-	-	-	**1**	-	-	**1**	-
Septoria convolvuli	-	-	-	-	-	-	-	**1**	-	-	-
Septoria cucubali	**1**	-	-	**1**	-	**1**	-	**1**	-	**1**	-
Septoriella dactylidis	-	-	-	-	-	-	-	-	-	-	**1**
Sistotrema brinkmannii	-	-	-	-	-	**1**	-	-	-	-	-
Ustilaginoidea virens	-	-	-	-	-	-	-	**1**	-	-	-
Vishniacozyma heimaeyen-	-	-	-	-	-	-	**1**	-	-	-	-
Xylaria sp.	-	-	-	**1**	**2**	**1**	-	-	-	-	1
**Total no. of isolates**	**13**	**18**	**23**	**27**	**21**	**26**	**23**	**17**	**14**	**15**	**22**
**Total no. of taxa**	**9**	**8**	**12**	**13**	**12**	**19**	**12**	**13**	**8**	**13**	**16**
**No. of taxa per sample**	**0.7**	**0.4**	**0.5**	**0.5**	**0.6**	**0.7**	**0.5**	**0.8**	**0.6**	**0.9**	**0.7**

BE-Belgium, CZ-Czech Republic, DK-Denmark, FR-France, DE-Germany, IE-Ireland, IT-Italy, LT-Lithuania, NL-Netherlands, PO-Poland, GB-UK.

Table S7. OTUs assigned to respective taxonomic level with total numbers of OTU obtained from seeds and leaves by HTS in the culture independent assessments.

Kingdom (OTU)	Phylum (OTU)	Class (OTU)	Order (OTU)	Family (OTU)
Fungi (71)	Ascomycota (56)	Dothideomycetes (28)	Botryosphaeriales (2)	Botryosphaeriaceae (2)
				Capnodiaceae (2)
			Capnodiales (3)	Mycosphaerellaceae (1*)
			Dothideales (2)	Dothioraceae (1*)
			Myriangles (3)	Elsinoaceae (2*)
			Pleosporales (18)	Didymosphaeriaceae (1)
				Montagnulaceae (3)
				Leptosphaeriaceae (3*)
				Phaeosphaeriaceae (6*)
				Pleosporaceae (3*)
			Venturiales (2)	Venturiaceae (1*)
		Eurotiomycetes (9)	Chaetothyriales (3)	Chaetothyriaceae (1)
				Herpotrichiellaceaea (1*)
			Eurotiales (6)	Trichocomaceae (6)
			Verrucariales (1)	Verrucariaceae (1*)
		Lecanoromycetes (3)	Lecanorales (1)	Ramalinaceae (1)
			Teloschistales (2)	Teloschistaceae (2)
		Leotiomycetes (3*)	Erysiphales (1)	Erysiphaceae (1)
			Helotiales (3)	Helotiaceae (1*)
			_	Vibrisseaceae (1*)
		Pezizomycetes (1)	Pezizales (1)	Ascobolaceae (1*)
		Sordariomycetes (7*)	Diaporthales (2*)	Diaporthaceae (2*)
			Hypocreales (3*)	Clavicipitaceae (1*)
				Hypocreaceae (1*)
				Nectriaceae (1)
			Glomerellales (1)	Glomerellaceae (1)
			Melanosporales (1)	Ceratostomataceae (1)
			Sordariales (2)	Sordariaceae (1*)
		Taphrinomycetes (5*)	Taphrinales (5*)	Taphrinaceae (5*)
	Basidiomycota (11)		Polyporales (1)	Fomitopsidaceae (1)
			Agaricales (1*)	_
		Agaricomycetes (2)	Russulales	Peniophoraceae
		Agaricostilbomycetes (1)	Agaricostilboles (1*)	Agaricostilbaceae (1*)
			_*	Kondoaceae (*)
		Cystobasidiomycetes (1*)	_*	_*
		Exobasidiomycetes (3*)	Micostromatales (1)	Microstomataceae (1)
			Entylomatales (1)	Entylomataceae (1)

		Microbotryomycetes (1*)	Sporidiobolales (1)	Sporidiobolaceaea (1)
			_*	_*
		Tremellomycetes (4*)	Tremellales (3*)	Tremellaceae (1)
				Bulleraceae (1)
			Cystofilobasidiales (1*)	Cystofilobasidiaceae (1)
			_*	-*
	Chytridiomy-cota (1)	Chytridiomycetes (1)	Rhizophydiales (1)	_*
	Zygomycota (3)	Mucoromycetes (3)	Mucorales (2)	Mucoraceae (2)
			_*	Rhizopodaceae (1)
Chromista(3)	Oomycota (3)	Oomycetes (3)	Pythiales (3)	Pythiaceae (3)

* indicates unidentified reads are present in that level.

Table S8. Dominant fungal endophytes identified from seed taken from two sites, Roosky and Glasnevin using the HTS of plant material. OTUs highlighted in bold are shared among sites and considered core.

Seed endophytes	Roosky	Glasnevin
Aspergillus niger	1	1
Aspergillus nomiae	1	0
Aspergillus penicillioides	1	1
Cladophialophora chaetospira	1	0
Diplodia mutila	1	0
Harzia acremonioides	1	1
Mucor abundans	1	1
Paraconiothyrium brasiliense	1	0
Paracamarosporium hawaiiense	1	1
Penicillium brevicompactum	1	1
Rhizopus arrhizus	1	0
Sordaria fimicola	0	1
Talaromyces minioluteus	0	1
Volutella ciliata	1	1
